# Eliminating PD‐L1 on Dendritic Cell Extracellular Vesicles for Immunotherapy Potentiates Immune‐Mediated Tumour Rejection in Mice

**DOI:** 10.1002/jev2.70322

**Published:** 2026-06-30

**Authors:** Loes Teeuwen, Loïc Steiner, Chantal Reinhardt, Annemarijn Offens, Jesse E. Kuipers, Daniel Martínez‐Martínez, Jules Mazouin, Benedict J. Chambers, Gözde Güçlüler Akpinar, Susanne Gabrielsson

**Affiliations:** ^1^ Division of Immunology and Respiratory Medicine, Department of Medicine Solna Karolinska Institutet Stockholm Sweden; ^2^ Department of Clinical Immunology and Transfusion Medicine Karolinska University Hospital Stockholm Sweden; ^3^ Center for Molecular Medicine (CMM) Karolinska University Hospital Stockholm Sweden; ^4^ Department of Medicine Huddinge, Center for Infectious Medicine Karolinska Institutet Stockholm Sweden; ^5^ Department of Microbiology, Tumor and Cell Biology Karolinska Institutet Stockholm Sweden

## Abstract

Extracellular vesicles (EVs) are emerging as promising vehicles for cancer immunotherapy, yet the molecular determinants of their immunogenicity remain poorly defined. While PD‐L1 expression on cancer‐derived EVs has been shown to suppress immune responses, its role on immunotherapeutic EVs remains unexplored. In this study, we investigated how eliminating PD‐L1 from antigen‐loaded bone marrow‐derived dendritic cell (BMDC) EVs affects the efficacy of EV‐based cancer vaccines.

We generated and characterized ovalbumin (OVA)‐loaded BMDC EVs from wild‐type (WT) and PD‐L1^−/−^ C57BL/6 mice. EVs were administered intravenously into WT mice in immunization experiments and in therapeutic and prophylactic B16 OVA‐secreting melanoma models. Immune responses were assessed by flow cytometry, ELISpot, and ELISA, and tumour growth was monitored.

Proteomic analysis confirmed high EV purity and similar protein profiles between WT and PD‐L1^−/−^ EVs, with PD‐L1 being the major difference. Functionally, PD‐L1^−/−^ EVs induced significantly stronger anti‐tumour responses in vivo, particularly in the prophylactic setting. Mice treated with PD‐L1^−/−^ EVs showed increased CD8^+^ T cell tumour infiltration, enhanced IFNγ secretion, and higher tumour rejection rates compared to WT EVs (72.7% vs. 37.5%). Additionally, the frequency of tumour‐infiltrating, antigen‐specific CD8^+^ T cells was significantly higher in PD‐L1^−/−^ EV‐treated mice.

In summary, BMDC‐derived EVs loaded with antigen are potent immune stimulators, and removal of immune checkpoint molecules such as PD‐L1 further enhances their immunogenicity. These findings support the development of engineered EVs as improved platforms for cancer immunotherapy.

## Graphical abstract

Extracellular vesicles (EVs) for immune therapy of cancer have both direct and indirect effects on immune cells. When taken up, the loaded antigen is presented by the host's dendritic cells (indirect). In this case, EV surface molecules like MHC‐peptide complexes on EVs are of minor importance. Here we show that removal of PD‐L1 from the surface of EVs increases their therapeutic potency, which demonstrates that in addition to the indirect effects, direct receptor‐ligand interactions between cells and EVs influence the therapeutic potency of EVs.

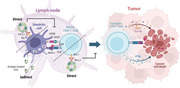

## 1 Introduction

Since the discovery of the immune system's role in cancer, immunotherapy has emerged as a promising treatment strategy (Esfahani et al. 2020; Waldman et al. 2020). Various forms of therapies are under investigation, including chimeric antigen receptor T cells, adoptive cell transfer, antibody therapies, and cancer vaccines. Among these approaches, antibodies that block immune checkpoint molecules such as PD‐1 and CTLA‐4 have shown to re‐activate anti‐tumoural immune responses (Ishida et al. 1992; Leach et al. 1996). This insight led to the development of monoclonal antibodies targeting PD‐1, its ligand PD‐L1, and CTLA‐4, which are now widely used in clinical settings (He and Xu 2020). However, not all patients respond, and adverse effects can be significant due to a general activation of the immune response (Fares et al. 2019; Martins et al. 2019).

Therapeutic cancer vaccines aim to stimulate tumour‐specific immune responses, particularly by activating cytotoxic T lymphocytes (CTLs). One strategy involves using antigen‐loaded dendritic cells (DCs) as live vaccines (Santos and Butterfield 2018). These DCs can migrate to lymph nodes and activate antigen‐specific CD8^+^ T cells to differentiate into CTLs but their effectiveness depends on their viability, migration, and activation, which can be modified by an immunosuppressive tumour microenvironment. To overcome these limitations, extracellular vesicles (EVs) derived from DCs are being explored as an alternative approach (Fernández‐Delgado et al. 2020; Markov et al. 2019; Xia et al. 2022).

EVs are lipid bilayer‐enclosed nanoparticles secreted by all cells (Bebelman et al. 2018; Raposo and Stoorvogel 2013; van Niel et al. 2018). They carry lipids, proteins, and nucleic acids reflective of their parent cells and are found in most biofluids. DC‐EVs can theoretically present both antigenic peptides via MHC class I and II molecules and intact antigens, along with co‐stimulatory signals. This enables them to activate naïve CD8^+^ T cells either directly or indirectly through antigen‐presenting cells (APCs) (Hiltbrunner et al. 2016; Markov et al. 2019; Meng et al. 2025; Offens et al. 2025; Pitt et al. 2014;). However, studies using MHC knockout or mismatched EVs have demonstrated that the primary mechanism for activating CD8^+^ T cells in vivo is through EV uptake followed by antigen presentation by APCs, rather than direct EV interaction with T cells (Hiltbrunner et al. 2016). Preclinical studies have demonstrated the potential of antigen‐loaded DC‐derived EV as cancer vaccines, though clinical trials have so far only confirmed their safety and feasibility (Besse et al. 2016; Escudier et al. 2005; Larssen et al. 2019; Markov et al. 2019; Morse et al. 2005; Veerman et al. 2023). While PD‐L1 on cancer derived EVs has been shown to suppress immune responses, the importance of this molecule in therapeutic EVs remains unclear, highlighting the need for further investigations to optimize EV‐based immunotherapy (Chen et al. 2018; Liu et al. 2022; Ma et al. 2025; Poggio et al., 2019).

In this study, we aimed to investigate the importance of PD‐L1 on therapeutic EVs by eliminating PD‐L1 from antigen‐loaded bone marrow‐derived dendritic cell (BMDC) EVs. Using EVs produced by BMDCs from wild‐type (WT) or PD‐L1 knockout (PD‐L1^−/−^) C57BL/6 mice, we evaluated immune responses, including T‐ and B‐cell responses specific to the model antigen ovalbumin (OVA), and anti‐tumour responses using both immunization and tumour models. We report that while EVs without PD‐L1 hardly induce stronger immune responses in immunization models, in tumour models, they are significantly more effective at reducing tumour growth, promoting T cell activation and intratumoural T cell infiltration compared to WT EVs.

## 2 Materials and Methods

### 2.1 Mice

Mice were used as donors for the generation of BMDC cultures, and as recipients for in vivo experiments monitoring the modulation of the immune response by EV administration. C57BL/6 (Taconic, Tornbjerg, Denmark), C57BL/6 PD‐L1^−/−^ (or B7‐H1‐KO) (kindly provided by Gennadiy Zelinsky, University Hospital Essen) and C57BL/6 CD45.1^+^OT‐I (kindly provided by Carmen Gerlach, Karolinska Institutet) mice were bred and kept under specific pathogen‐free conditions at the Karolinska Institutet's animal facility (David et al. 2019; Dong et al. 2004). Except for CD45.1^+^OT‐I, mice in experiments were female. All mice were between 7 – 12 weeks old when used in experiments. Ethical approval for the study was obtained from the Stockholm Animal Ethics Committee (8648‐2020 and 12650‐2025). Mice were acclimatized for at least 5 days prior to the start of the experiments.

### 2.2 BMDC EV Generation

To obtain large numbers of BMDCs for EV generation, BMDCs were differentiated through the addition of GM‐CSF and IL‐4 to the culture medium. Bone marrow cells were isolated from C57BL/6 WT and PD‐L1^−/−^ mice on day 0. Erythrocytes were lysed with ACK lysis buffer (0.8% NH_4_Cl (w/v), 0.1% KHCO_3_ (w/v), 0.1 mM EDTA) and the remaining cells were cultured in complete RPMI (SH30255.01, Cytiva) medium (10% heat‐inactivated FCS [56°C, 30 min] (HI‐FCS; A5257601, Gibco), 1 mM sodium pyruvate (11360‐039 Gibco), 500 µg/mL Penicillin‐Streptomycin (SV30010, Cytiva), 2 mM L‐Glutamine (SH30034.01 Cytiva), 50 µM β‐Mercaptoethanol (M7522 Sigma‐Aldrich), 10 ng/mL GM‐CSF (576308, BioLegend), 2 ng/mL murine IL‐4 (214‐14‐1MG, ReproTech)) at 37°C, 5% CO_2_ at a density of 115,000 cells/cm^2^ and 2.5 mL complete medium per 10^6^ cells. On day 3, the same volume of complete medium was added. For antigen‐loaded EVs, 300 µg/mL of the model antigen OVA (A5503‐25G, Sigma‐Aldrich) was added to the culture on day 6.

On day 7, loosely attached cells were transferred to EV‐depleted complete medium supplemented with 30 ng/mL LPS (L6529, Sigma‐Aldrich) for activation of the BMDCs at a density of 285,000 cells/cm^2^ and 5 mL EV‐depleted complete medium per 10^6^ cells. EV‐depleted medium was prepared by ultracentrifugation of 30% HI‐FCS in RPMI at 70,000 x g_average_, 4°C, ≥16 h, followed by 0.22 µm filtration (99500, TPP). On day 9, conditioned supernatant and loosely attached cells were collected and the supernatant was cleared by sequential centrifugation (300 x g, 10 min; 3000 x g, 30 min, both at 4°C), filtered through a 0.45 µm cut‐off filter (83.3941.100, SARSTEDT) and stored at 4°C until further processing.

### 2.3 EV Isolation

The filtered and pre‐centrifuged supernatant from the day 9 BMDC culture was purified and concentrated for EV isolation using tangential flow filtration (TFF, KrosFlo) with an mPES/300 kD 370 cm^2^ column (D06‐E300‐05‐N, Repligen). The filtered supernatant was centrifuged in an Optima L‐100 XP ultracentrifuge (Beckman Coulter) at 16,500 x g at 4°C for 30 min. The supernatant was then filtered through a 0.22 µm filter (99722, TPP). Subsequently, the filtered supernatant was subjected to two rounds of ultracentrifugation at 100,000 x g_average_ at 4°C for 2 h. The resulting pellet was carefully resuspended in a small volume of PBS (100–200 µL). The protein content of the EV suspension was quantified using protein DC assay according to the manufacturer's instructions (500‐0112, Bio‐Rad) and the OVA‐content was assessed with an OVA‐specific ELISA. Unless stated otherwise, EV OVA‐content of groups that were used in experiments was comparable. The EVs were stored at −80°C until further use.

### 2.4 Nanoparticle Tracking Analysis

Nanoparticle Tracking Analysis (NTA) was used to determine the size distribution and diameter of EVs. Samples were diluted in 0.22 µm filtered PBS to an ideal particle concentration of 30 to 120 particles per frame. Samples were recorded in five 30‐s videos under the following conditions using NTA3.0 software: perfusion rate 50, camera level 13, detection threshold 4, Nanosight LM10‐HSGF system (LM14 particle viewing unit, CMOS camera, 488 nm laser, syringe pump).

### 2.5 Negative Stain Transmission Electron Microscopy (TEM)

A 3 µL aliquot of each EV sample was placed on a glow‐discharged, carbon‐coated grid for 3 min. Excess liquid was removed with filter paper. The grid was then rinsed with MiliQ water and excess liquid was removed with filter paper. Subsequently, the grid was stained with uranyl acetate in water for 7 s. After air‐drying, the samples were placed into a Hitachi HT 7700 electron microscope. Images were captured using a Veleta camera (Olympus) at an electron beam power of 80 kV.

### 2.6 Proteomic Analysis

EV samples were analysed by label free quantification. The EVs were dissolved in lysis buffer (2% SDS, 50 mM HEPES pH 7.6, 1 mM DTT). Protein digestion (LysC and trypsin, sequencing grade modified, Pierce) was performed using a modified protocol for SP3 protein clean‐up (Moggridge et al. 2018) followed by SCX peptide clean‐up. Each sample was separated using a Thermo Scientific Dionex nano LC‐system in a 3 h 5%–40% ACN gradient coupled to Thermo Scientific High Field QExactive. The software Proteome Discoverer versus 1.4 including Sequest‐Percolator for improved identification was used to search the *Mus musculus* Uniprot database for protein identification, limited to a false discovery rate of 1%.

Protein abundance in different EVs was quantified based on MS1 precursor ion intensities (integrated peak areas) and analysed in R (version 4.5.0, R Foundation for Statistical Computing). Data processing, statistical analysis and visualization were performed using the following R packages: tidyverse, limma, imp4p, DEP, ggplot2, pheatmap, and ggbeeswarm. Proteins were filtered and included in the differential abundance analysis if detected in at least 2 out of 3 replicates in at least one group. Samples were normalized using cyclic loess algorithm. In cases where a protein was entirely absent in one group, missing values were imputed with random values drawn from a gaussian distribution centred around the lower expression levels of all other proteins using impute.pa() with q.min = 0.025, q.norm = 3 and distribution = ‘unif.’ Gene set enrichment analysis was conducted using the GSEA software v4.3.3 from the Broad Institute (https://www.gsea‐msigdb.org/gsea/index.jsp). The gene sets MH (Hallmark gene sets), M2 (curated gene sets) and M3 (regulatory target gene sets) were used as reference gene sets. Parameters for GSEA include 1000 permutations of gene set and Signal2Noise as metric for ranking genes. Due to the low number of proteins, gene sets containing <3 or >500 genes/proteins were excluded from analysis.

Enrichment analysis of differentially abundant proteins (CD274 was removed from the list to search for systematic changes other than the knock out) in biological pathways or functional gene categories was performed using Enrichr (https://maayanlab.cloud/Enrichr/) (Kuleshov et al. 2016). Enrichment results were visualized in R using the enrichR package.

### 2.7 Splenocyte Binding

EVs were labelled with 1 µM celltracker deep red (CTDR, C34565, Thermo Fisher) for 1 h at 37°C. The labelled EVs were diluted in PBS and concentrated using 100 kDa spin filters (UFC9100, Sigma Aldrich) to remove excess unbound dye. A dye control was generated by performing the same steps without EVs. Subsequently, 2 × 10^5^ C57BL/6 mouse splenocytes in medium (RPMI, 10% HI‐FCS, 2 mM/L L‐glutamine, 1X penicillin‐streptomycin) were incubated with increasing concentrations of EVs (0 µg, 0.5 µg, 1 and 2 µg) for 1 h at 4°C. Splenocytes were stained and analysed by flow cytometry as described below.

### 2.8 In Vitro Activation of Flt3L‐BMDCs by EVs

To test in vitro activation of steady state like BMDCs by EVs, BMDCs were differentiated through addition of Flt3L to the culture medium. Bone marrow cells were isolated from C57BL/6 WT mice on day 0. Erythrocytes were lysed with ACK lysis buffer and the remaining cells were cultured in base RPMI medium (10% HI‐FCS, 1 mM sodium pyruvate, 500 µg/mL Penicillin‐Streptomycin, 1 mM L‐Glutamine, 50 µM β‐Mercaptoethanol) supplemented with 200 ng/mL Flt3L (250‐31L, ThermoFisher) at 37°C, 5% CO_2_ at a density of 6 × 10^6^ cells/well in 6‐well plates in 4 mL medium. On day 7, loosely attached cells were harvested by gentle rinsing with pre‐warmed RPMI, and all subsequent handling was performed at room temperature with pre‐warmed media to preserve DC functionality.

Harvested BMDCs were seeded at 50,000 cells/well in 200 µL base RPMI medium without Flt3L in 96‐well round‐bottom plates and stimulated for 24 h at 37°C with the following conditions: PBS (negative control), LPS alone (100 ng/mL; positive stimulation control), LPS + OVA (2 µg/mL; positive OT‐I T‐cell activation control), OVA‐loaded WT EVs (50 µg/mL) and OVA‐loaded PD‐L1^−/−^ EVs (50 µg/mL). After 24 h, activated BMDCs were either used for OT‐I T‐cell co‐culture or stained and analysed by flow cytometry as described below.

### 2.9 OT‐I T‐Cell Activation Assay

Spleens from CD45.1^+^ OT‐I mice were homogenized through a 100 µm cell strainer (431752, CORNING). After erythrocyte lysis with ACK buffer, cells were resuspended in MACS buffer (PBS, 1% HI‐FCS, 2 mM EDTA). CD8^+^ T‐cells were enriched by negative selection using the Miltenyi Pan T‐cell Isolation Kit (130‐096‐535, Miltenyi Biotec) according to the manufacturer's instructions. Purified T‐cells were resuspended in T‐cell medium (RPMI, 10% HI‐FCS, 2 mM L‐Glutamine, 1 mM sodium pyruvate, 1X NEAA (11140050, Gibco), 50 µM β‐Mercaptoethanol, 500 µg/mL Penicillin‐Streptomycin) and purity confirmed by flow cytometry. CD8^+^CD4^−^TCRb^+^ cells constituted >87% of live cells in all experiments.

Purified CD45.1^+^ OT‐I T‐cells were then co‐cultured with EV‐activated CD45.2^+^ Flt3L‐BMDCs: after 24 h of activation of BMDCs, they were washed with complete T‐cell medium and resuspended in 100 µL/well. OT‐I T‐cells were added at 100,000 cells/100 µL T‐cell medium per well and co‐cultured for 18 h at 37°C to assess T‐cell activation capacity of the activated BMDCs.

### 2.10 Immunization Model

7‐ to 8‐week‐old female C57BL/6 mice were intravenously injected on day 0 and 14 with 40 µg of OVA‐loaded BMDC EVs from WT or PD‐L1^−/−^ mice, or with 100 µL PBS. Mice were sacrificed on day 21. Spleens were resected and stored in RPMI at 4°C until further processing. Blood was sampled from the inferior vena cava into microtainer collection tubes (365968 BD Bioscience) and stored at RT until further processing.

### 2.11 B16F1 Secreting OVA Cell Line

B16F1 cells secreting OVA (B16sOVA) were cultured in a medium consisting of RPMI, 10% HI‐FCS, 2 mM L‐glutamine, 1X PEST and 400 µg/mL geneticin (SV30068.01, HyClone) for five days across two passages, maintained at 37°C with 5% CO_2_. B16sOVA cells were detached using 1X Trypsin‐EDTA solution (SLCC3240, SIGMA), centrifuged at 400 x g at 4°C for 5 min, and resuspended in sterile PBS for injection or in complete medium for ELISpot. The culture was tested for *Mycoplasma* contamination using the Lookout Mycoplasma PCR Detection Kit (MP0035, Sigma‐Aldrich) according to the manufacturer's instructions.

### 2.12 Tumour End Day and Survival Model

7‐ to 8‐week‐old female C57BL/6 mice were subcutaneously injected with 1 × 10^5^ B16sOVA on the right flank on day 0. On days 5 and 12, they were intravenously injected with 40 µg of BMDC EVs from WT or PD‐L1^−/−^ mice, or with 100 µL PBS. Tumour size and mouse weight were measured every 2–3 days. In tumour survival experiments, each mouse was sacrificed upon reaching a tumour size of >1000 mm^3^ (length × width × height), or upon ulceration/humane endpoint. In tumour end day experiments, all mice were sacrificed when the PBS group reached >1000 mm^3^. Tumour‐draining lymph nodes (TDLN), spleens, and tumours were resected and stored in RPMI at 4°C until further processing. Blood was sampled from the inferior vena cava into microtainer collection tubes (365968, BD Bioscience) and stored at RT until further processing.

### 2.13 Prophylactic Tumour Model

7‐ to 8‐week‐old female C57BL/6N mice were intravenously injected on day 0 and 14 with 40 µg of OVA‐loaded BMDC EVs from WT or PD‐L1^−/−^ mice, or with 100 µL PBS. For PD‐L1^−/−^ EVs, two doses (2×12 µg and 2×40 µg EVs) were used to account for a potential effect from the differences in OVA‐content between WT and PD‐L1^−/−^ EVs in these batches. Subsequently, on day 21, mice were subcutaneously injected with 1 × 10^5^ B16sOVA cells in the right flank. Tumour size and mouse weight were measured every 2–3 days. Each mouse was sacrificed upon reaching a tumour size of1000 mm^3^, or upon ulceration/humane endpoint. TDLNs, spleens, and tumours were resected and stored in RPMI at 4°C until further processing. Blood was sampled from the inferior vena cava into microtainer collection tubes (365968, BD Bioscience) and stored at RT, for at least 30 min, until further processing.

### 2.14 Tissue Processing

Collected blood was centrifuged at 13,000 x g for 10 min to isolate serum, which was then stored at −20°C. Tumours were cut into 2–3 mm pieces using scissors and incubated for 30 min at 37°C in 1 mL RPMI with 0.5 Wünsch U/mL Liberase TL (05401020001, Roche). Tumours, spleens, and TDLNs were passed through a 100 µm cell strainer using a syringe plunger and RPMI to flush the strainer. Cells were centrifuged at 300 x g for 10 min. Tumour and spleen cell pellets were resuspended in 1 mL ACK and incubated for 90 s at RT. 20 mL RPMI was added to stop lysis, and cells were centrifuged at 300 x g for 10 min. Tumour and spleen cell pellets were resuspended in RPMI, and cells were counted using the Countess 3 automatic cell counter (Invitrogen). TDLN cells were used without further processing.

### 2.15 Flow Cytometry

Antibodies were added to BMDCs and incubated for 30 min at 4°C in the dark. The samples were washed with PBS and centrifuged again at 400 x g at 4°C for 5 min, then resuspended in PBS.

The presence of surface proteins on EVs was evaluated using bead‐based flow cytometry. Briefly, SPHERO Streptavidin Magnetic Particles (SVMS‐40‐10) were coated with biotinylated anti‐CD9 (clone EM‐04, Biosite Flow) and incubated with 2 µg of EVs per staining. The beads were washed, and staining was performed using final antibody concentrations of 1 µg/mL for 30 min at 4°C. The beads were washed and resuspended in PBS.

Before staining viability and activation markers on Flt3L BMDCs and OT‐I T‐cell co‐culture, cells were incubated with mouse Fc block (553142, BD Pharmingen, 1:100 in PBS) for 30 min at 4°C. Stainings were performed at 4°C for 30 min in Brilliant Stain Buffer (00‐4409‐75, Invitrogen). To assess viability of BMDCs without OT‐I T‐cell co‐culture, a second step staining of AnnexinV was performed in AnnexinV binding buffer (422201, BioLegend) which was used in all subsequent washes and resuspension. For viability, BMDC alone and OT‐I T‐cell co‐cultures were resuspended in 200 µL AnnexinV binding buffer or PBS, respectively, containing 3 µM DAPI (422801, BioLegend).

Isolated cells from spleens, tumours and TDLNs were stained and incubated for 30 min at 4°C with 100 µL blocking solution containing LiveDead staining (L34962 Invitrogen, 1:1000), mouse Fc block, with or without pentamer staining (F093‐2B‐E‐93‐H‐2Kb‐SIINFEKL, Pentamer R‐PE, PROIMMUNE, binds the MHC‐I:peptide complex of OVA‐specific CD8^+^ T cells, 1:50). After washing with PBS, the cells were stained with surface antibody panels for 30 min at 4°C. All cells were washed and resuspended in PBS.

Flow cytometric analysis of BMDC phenotyping, EV phenotyping and the EV binding experiment was acquired on a BD FACSCanto II flow cytometer. Cell phenotyping for activation‐, immunization‐ and tumour experiments were acquired with a BD LSR Fortessa flow cytometer. Data analysis was performed using FlowJo v10.10 Software (BD Life Sciences). Employed antibodies and dyes are listed in Table S1. All antibodies, unless state otherwise, were diluted to final working concentration of 1 ng/mL.

### 2.16 ELISpot

ELISpot was used to analyse IFNγ production upon restimulation of splenocytes. PVDF plates were coated with IFNγ capture antibody (1:200, 3321‐3‐250, Mabtech) in PBS overnight at 4°C. Excess uncoated antibody was removed by washing with PBS. Plates were then blocked with blocking solution (RPMI culture media + 10% HI FCS) for 2 h at 37°C. Next, 2 × 10^5^ cells were incubated with different stimuli: 2 µg/µL concanavalin A (11028‐71‐0, Sigma‐Aldrich), 2 µg/µL CD8 peptide OVA SIINFEKL (SP‐O257‐1, Innovagen), or 2 × 10^5^ B16sOVA. After stimulation, plates were washed with PBS and biotinylated anti‐IFNγ detection antibodies were added (3321‐6‐250, MabTech, 1:1000) at RT for 2 h. Plates were then washed with PBS and streptavidin‐alkaline phosphatase (SA‐ALP, 3310‐10‐1000, MabTech, 1:1000) was added at RT for 1 h. The plates were washed, and substrate was added (BCIP/NBT‐plus, 3650‐10, MabTech). All plates were read with the AID iSpot FluoroSpot reading system and analysed with AID EliSpot software (Autoimmun Diagnostika).

### 2.17 ELISA

All washing steps consisted of 5 times washing with PBS + 0.05% Tween 20 using a plate washer (BioTek AH diagnostics).

Ova‐specific ELISA was performed. Briefly, Nunc Maxisorp flat‐bottom plates were coated with 5 µg/mL EVs, or an OVA standard overnight at 4°C. The next day, the plates were washed and blocked with 5% BSA in PBS for 2 h at RT, followed by incubation with primary anti‐OVA antibody (clone 3G2E1D9, LSbio, 1:1000) in PBS for 1 h at RT. The plates were washed and incubated with anti‐mouse IgG HRP (1036‐05, Southern Biotech, 1:2000) in PBS for 1 h at RT. Lastly, after washing, HRP substrate (3652‐F10, Mabtech) was added. The reaction was stopped with 1 M H_2_SO_4_. The absorbance was read at 450 nm.

To measure anti‐OVA titers in serum, ELISA plates were coated with 50 µL/well of OVA‐specific (10 µg/mL) or IgG antibody (3 µg/mL) and incubated overnight at 4°C. The plates were washed, then 50 µL of diluted serum was added to the designated wells and incubated at RT for 2 h. After washing, detection antibodies for IgG‐AP (1030‐04, Southern BioTech, 1:2000), IgG1‐AP (ASB‐107004, Biosite, 1:2000), or IgG2c‐AP (ASB‐107904, BioSite, 1:2000) were added and incubated at RT for 1 h. Subsequently, alkaline phosphatase substrate (P4744‐1G, Sigma Aldrich) was added and kept in the dark at RT. Absorbance was measured at 405 nm after 25 min for total IgG, 45 min for total OVA‐IgG, and 30 min for IgG1 and IgG2c. In both ELISAs, absorbance was measured using an ELISA plate reader (Thermo Scientific Varioskan Lux).

### 2.18 Statistical Tests

Unless specified, data were analysed using one‐way ANOVA with Tukey's test for multiple comparisons while comparing different groups. Statistical analyses were performed for all relevant pairwise comparisons, and statistical significance is indicated for significant differences (*p* < 0.05) in the figures. Survival differences were tested using Mantel‐Cox test.

## 3 Results

### 3.1 WT and PD‐L1^−/−^ EVs Show Similar Surface Marker Pattern, Size and Morphology

**FIGURE 1:**
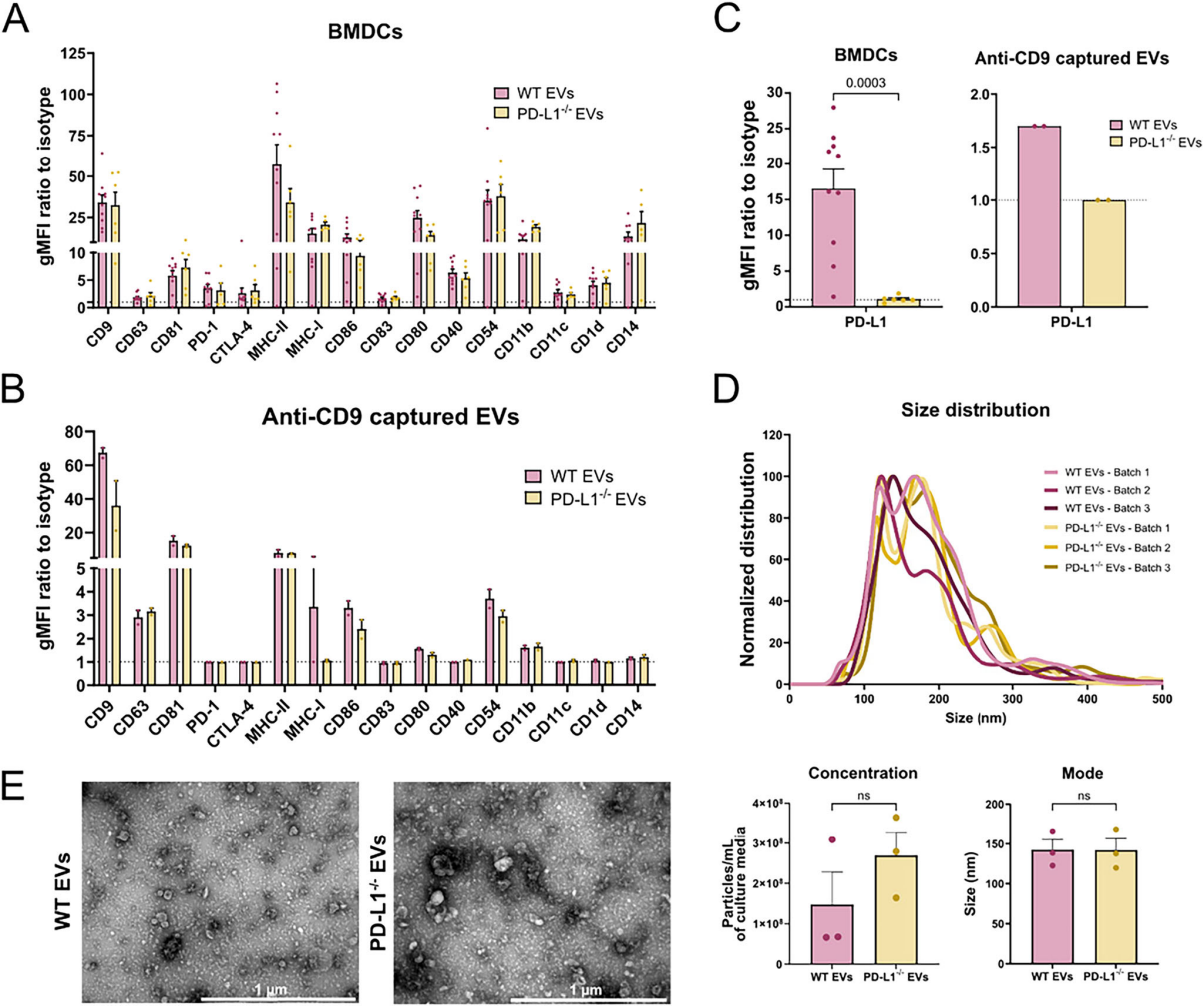
WT and PD‐L1^−/−^ EVs show similar surface marker pattern, size and morphology. (A–C) Flow cytometric analysis of surface protein expression, displayed as ratio of geometric mean fluorescence intensity (gMFI) in staining to gMFI of isotype control on (A) BMDCs on day 9 of culture (*n* = 6–10 independent biological replicates) on (B) CD9‐bead captured EVs (*n* = 2 independent biological replicates) and (C) for PD‐L1. (D) Nanoparticle tracking analysis (NTA) of EV isolates depicting size distribution, particle concentration and particle mode size (*n* = 3 independent biological replicates). (E) Negative stain TEM pictures of EV isolates. Scale bar is 1 µm. Data are shown as mean ± SEM. Unpaired *t*‐tests between each protein with multiple comparison correction using the FDR approach, Q = 5% (A and B) and unpaired *t*‐test (C and D). Only statistically significant differences (*p* < 0.05) are indicated.

EVs were isolated from OVA‐fed BMDCs derived from WT and PD‐L1^−/−^ mice using tangential flow filtration followed by differential ultracentrifugation. We first assessed the expression of 19 surface proteins on EV‐donor cells post antigen‐loading and activation at the time of EV harvesting (Figure 1A). BMDCs showed low expression of CD63, PD‐1, CTLA‐4, CD83, and CD11c; moderate expression of CD81, CD40, and CD1d; and high expression of CD9, MHC‐I, MHC‐II, CD86, CD80, CD54, CD11b, and CD14.

EVs are nanoscale particles that approach the lower detection limit of conventional flow cytometry. To enable analysis, EVs were captured on anti‐CD9‐coated beads and assessed in bulk (Figure 1B). Note that this approach will exclude CD9^−^ EVs present in the EV isolate. Compared to their parental BMDCs (Figure 1A), CD9^+^ EVs exhibited a distinct surface marker profile. As expected, EV markers such as CD9, CD63 and CD81 showed high intensity staining on EVs, as well as MHC‐II, CD86 and CD54. Other markers that were expressed on BMDCs were not found on EVs, such as CD14, CD11b or CD80. In both BMDCs and CD9^+^ EVs, some variations in surface marker expression were observed between the groups; however, these differences were not statistically significant. Thus, both EV groups share similar surface marker expression. PD‐L1 was absent on PD‐L1^−/−^ BMDCs and highly expressed on WT BMDCs (*p* = 0.0003), confirming successful knockout (Figure 1C). Similarly, WT EVs showed a clear PD‐L1 signal, which was absent in PD‐L1^−/−^ EVs. Nanoparticle tracking analysis (NTA) revealed no significant differences in size distribution, concentration, or mode size between EV types (Figure 1D). Similarly, negative‐stain TEM showed comparable morphology, consistent with EVs (Figure 1E).

In conclusion, antigen‐loaded WT and PD‐L1^−/−^ BMDC‐derived EVs show a similar phenotype and share typical EV characteristics.

### 3.2 Proteomic Profiles of EVs Derived From WT and PD‐L1^−/−^ BMDCs Show No Overt Immune‐Related Differences

**FIGURE 2:**
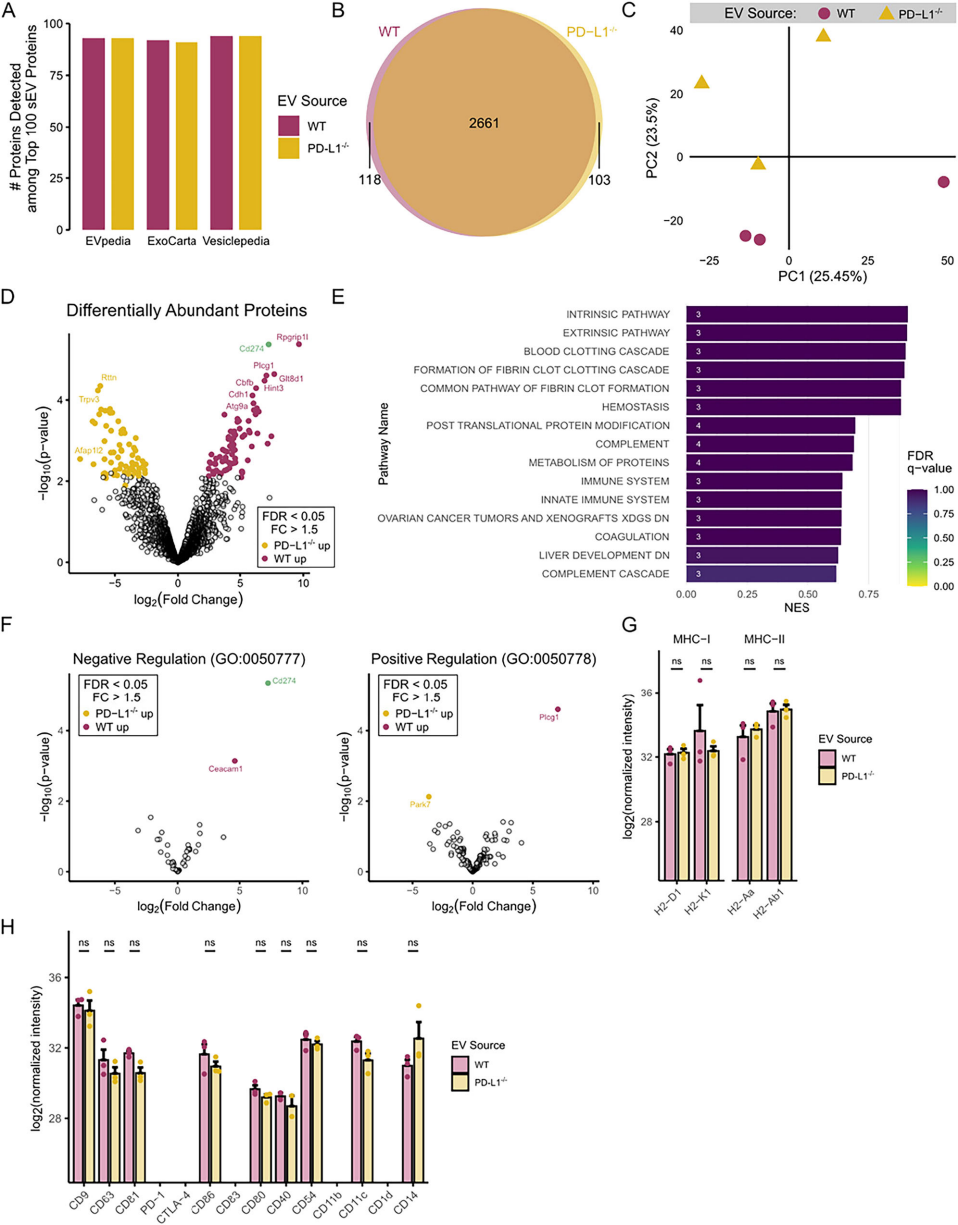
Proteomic profiles of EVs confirmed knockout of PD‐L1 (CD274), but no other overt changes in immune‐related pathways between EVs from PD‐L1^−/−^ and WT mice. (A–H) Proteomic profile of EVs from WT and PD‐L1^−/−^ BMDCs was analysed by LC‐MS/MS (*n* = 3 independent biological replicates). (A) Number of proteins, out of the top 100 detected proteins in three different EV databases, that were detected in WT resp. PD‐L1^−/−^ EVs. (B) Overview of robustly detected proteins (detected in at least 2/3 replicates). (C) Principal component analysis of proteins detected in all samples. (D) Volcano plot showing differentially abundant proteins, defined by a threshold of fold change (FC) > 1.5 and an adjusted *p*‐value (FDR) < 0.05. PD‐L1 (CD274) is depicted in green for clarification. (E) Gene set enrichment analysis (GSEA) showing the normalized enrichment score (NES) for the 15 gene sets with the highest NES. Bar colour indicates the adjusted *p*‐value (FDR q value), and white numbers within the bars represent the number of detected proteins per gene set. (F) Volcano plot from (D) filtered for the GO‐terms GO:0050777 (Negative Regulation of Immune Responses) and GO:0050778 (Positive Regulation of Immune Responses). (G) Comparison of protein abundances of MHC‐I and MHC‐II molecules on EVs as determined by MS. (H) Comparison of protein abundances of BMDC and EV markers on EVs as determined by MS. (G and H) Data are shown as mean ± SEM on the log_2_ scale. Data were analysed using *t*‐tests with multiple testing correction using the Benjamini‐Hochberg method.

To further compare EVs derived from WT and PD‐L1^−/−^ BMDC EVs, we performed proteomic analysis by label free liquid chromatography tandem mass spectrometry (LC‐MS/MS) on three independent batches of both EV types. To assess the quality of the EV isolate, which for MS analysis was not further purified with anti‐tetraspanin beads as was done for flow cytometry, we analysed our MS data for the presence of proteins described in three different EV databases (Figure 2A). Indeed, we found most of the top 100 described proteins of each database in both EV groups, confirming the quality of our EV isolates. We then analysed the distribution of robustly detected proteins (detected in at least 2 out of 3 replicates in at least one EV group) between WT and PD‐L1^−/−^ EVs (Figure 2B). The majority of proteins was detected in both EV groups. To assess sources of variance within the proteomic dataset, we performed principal component analysis (PCA) (Figure 2C). PC1 accounted for 25.45% and PC2 for 23.5% of the total variance. Notably, WT and PD‐L1^−/−^ EVs were mostly separated along PC2, while biological replicates of WT EVs showed a broader distribution along PC1. This indicates that sample‐to‐sample variability, due to biological or technical variation, contributes substantially to the overall variance, with differences between WT and PD‐L1^−/−^ EVs being secondary on PC2. Similarly, hierarchical clustering failed to cluster all WT and PD‐L1^−/−^ samples together (Figure S1A). This analysis indicated similar overall proteomic composition of the two EV types, however some proteins were found to be differentially abundant on WT and PD‐L1^−/−^ EVs (Figure 2D). Among the strongest upregulated proteins in WT EVs was PD‐L1 (CD274), which was completely absent in PD‐L1^−/−^ EVs, thus confirming again presence of PD‐L1 in WT EVs and successful knockout.

To investigate whether changes in the proteomic composition of the EVs were due to biological differences between WT and PD‐L1^−/−^ cells, or rather the stochastic nature of the MS analysis and variation between individual EV batches, we performed gene set enrichment analysis using the software tool GSEA (Figure 2E and Figure S1B) and the web‐based Enrichr platform (Figure S1C). Indeed, no pathways were significantly enriched, as all GSEA results showed *p*‐values close to 1. With Enrichr, only few, seemingly non‐immune immune related pathways were enriched, such as brain cell necrosis in vascular dementia. Furthermore, to confirm that the main changes between WT and PD‐L1^−/−^ EVs when analysed in the context of cancer immunotherapy is the missing PD‐L1, we filtered the data for proteins involved in negative and positive regulation of immune responses (Figure 2F). Indeed, most proteins associated with these GO‐terms were not changed between the two EV groups, with the strongest exception being PD‐L1, followed by Plcg1, Ceacam1 and Park7. Last, to confirm the flow cytometry analysis on CD9^+^ EVs, we investigated the abundance of MHC molecules (Figure 2G) and BMDC markers (Figure 2H) in the MS proteomic data. This confirmed similar levels of MHC‐I and ‐II molecules on the EVs. Additionally, no difference in other BMDC or EV markers were observed using this method.

Altogether, while proteomic analysis showed some differences between WT and PD‐L1^−/−^ EVs, these in part reflected variability across biological replicates, and were not consistently associated with immune‐regulatory pathways. Therefore, the presence or absence of PD‐L1 represents the primary discerning factor between the two EV types.

### 3.3 PD‐L1^−/−^ EVs Induce Stronger BMDC Activation and T‐Cell Priming Than WT EVs

To investigate whether the absence of PD‐L1 would affect binding of EVs to different cell types, WT splenocytes were incubated for 1 h at 4°C with CTDR‐labelled EVs (Figure S2A,B). No significant differences were observed in binding of the two EV types to DCs, B cells, NK cells, CD4^+^ T cells, CD8^+^ T cells, Granulocytes, Monocytes or Macrophages.

**FIGURE 3:**
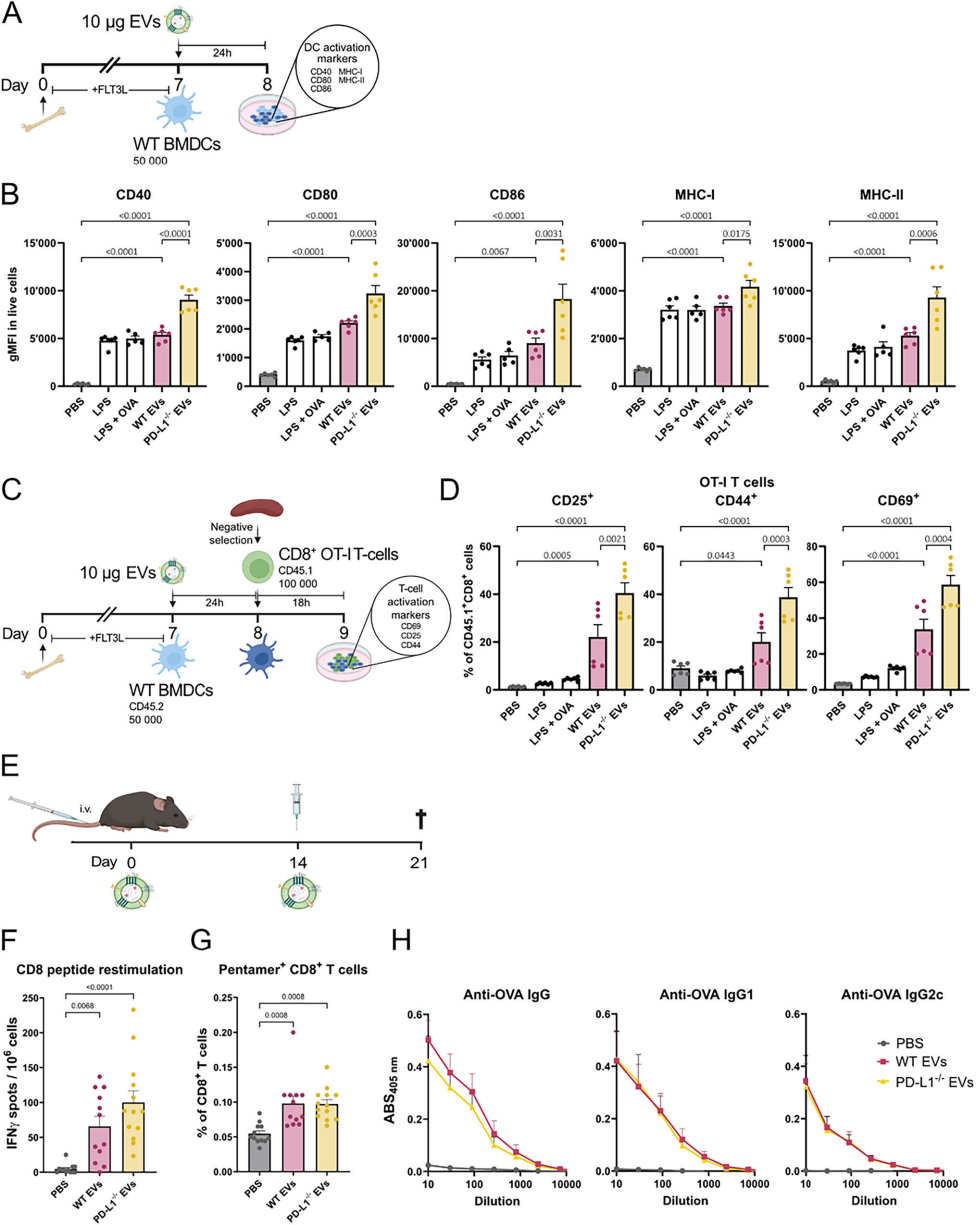
PD‐L1^−/−^ EVs induce stronger in vitro but similar in vivo immune responses. (A) Schematic representation of Flt3L‐differentiated BMDC in vitro activation by EVs. (B) Geometric mean of fluorescent intensity (gMFI) of the DC activation markers CD40, CD80, CD86, MHC‐I and MHC‐II in all live cells assessed by flow cytometry, after 24 h incubation with either PBS, LPS (100 ng/mL), LPS (100 ng/mL) + OVA (2 µg/mL), WT or PD‐L1^−/−^ EVs (50 µg/mL). (C) Schematic representation of in vitro OT‐I T cell activation by EV‐activated Flt3L‐differentiated BMDCs. (D) Frequency of OT‐I (CD45.1^+^) CD8^+^ T cells positive for the activation markers CD25, CD44 or CD69 assessed by flow cytometry, after 18 h of co‐culture with BMDCs activated as described in (A and B). Data represents pooled results from 2 independent experiments (B and D). (E) Schematic representation of in vivo immunization. (F) IFNγ ELISpot signal in splenocytes after restimulation with the OVA‐derived CD8^+^ T cell peptide SIINFEKL. (G) Frequency of OVA‐specific (Pentamer^+^) CD8^+^ T cells out of all CD8^+^ T cells (B220^−^ CD3^+^ CD8^+^) in the spleen, as assessed by flow cytometry. (H) ELISA for detection of OVA‐specific antibodies in serum. Pooled data from two independent experiments (*n* = 12 biological replicates). Data are shown as mean ± SEM. Statistical analysis was performed using one‐way ANOVA with Tukey's test for multiple comparisons. Only statistically significant differences (*p* < 0.05) are indicated. Comparisons involving LPS and LPS/OVA controls are omitted from the graphs for clarity (B and D).

As previous work from our group has shown efficient induction of immune responses in vivo by therapeutic BMDC‐EVs independently of MHC‐molecules (Hiltbrunner et al. 2016), we hypothesized that APCs are the main targets of therapeutic EVs in this setting. We thus compared the ability of PD‐L1^−/−^ and WT EVs to activate DCs in vitro using Flt3L‐differentiated BMDCs as a model. Flt3L differentiation yields heterogenous DC subsets similar to those found in the spleen with strong antigen presentation capacity (Naik et al. 2007). BMDCs were incubated with EVs for 24 h and analysed via flow cytometry (Figure 3A). Interestingly, while both EV groups increased the expression levels of the activation markers CD40, CD80, CD86, MHC‐I and MHC‐II, PD‐L1^−/−^ EVs caused a more pronounced increase than WT EVs in all analysed markers (Figure 3B). Viability was not affected by PD‐L1 on EVs, as both EV types yielded similar proportions of live, early apoptotic and late apoptotic cells (Figure S2C,D). The frequency of CD11c^+^ cells was more strongly increased in BMDCs incubated with PD‐L1^−/−^ than WT EVs, but no significant differences in frequencies of pDCs, cDC1s or cDC2s were observed (Figure S2E).

To investigate this further, we assessed whether removal of PD‐L1 from OVA‐loaded EVs would subsequently increase the capacity of the BMDCs to promote T‐cell responses. We purified splenic T‐cells from CD45.1^+^OT‐I mice, which express a transgenic T‐cell receptor specific for the MHC‐I restricted SIINFEKL peptide of OVA, and co‐cultured them for 18 h with EV‐activated Flt3L‐differentiated CD45.2^+^BMDCs. We then assessed the activation of the T‐cells by flow cytometry (Figure 3C). Indeed, while BMDCs incubated with both EV groups resulted in a strong T‐cell response, the upregulation of the early T‐cell activation marker CD69, as well as of the T‐cell activation markers CD25 and CD44 was stronger with PD‐L1^−/−^ than with WT EVs (Figure 3D).

In summary, these results show that removal of PD‐L1 from OVA‐loaded EVs increases their capacity to activate BMDCs and enhances the ability of BMDCs to prime T‐cells.

### 3.4 PD‐L1^−/−^ and WT EVs Induce Similar Immune Activation in an Immunization Model

To assess the effect of PD‐L1 on EVs when inducing an immune response in vivo, we employed an immunization mouse model. WT mice received 40 µg of either WT or PD‐L1^−/−^ EVs on day 0 and day 14, and were sacrificed on day 21 (Figure 3E).

Both WT and PD‐L1^−/−^ EV immunizations induced a strong secretion of IFNγ in splenocytes upon restimulation with the OVA‐specific CD8^+^ T cell peptide SIINFEKL (*p* = 0.0068 and *p* < 0.0001, respectively; Figure 3F). This secretion of IFNγ indicates enhanced OVA‐specific T cell effector capacity upon EV treatment. The frequency of OVA‐specific CD8^+^ T cells, measured by flow cytometry using a SIINFEKL:MHC‐I pentamer, did not differ significantly between EV‐treated groups (Figure 3G, gating's Figure S3). No differences were detected in serum levels of OVA‐specific IgG antibodies across EV treatment groups (Figure 3H).

In conclusion, both EV groups were able to induce immune responses of similar magnitudes against the model antigen OVA.

### 3.5 Therapeutic PD‐L1^−/−^ EVs Induce a Strong Anti‐Tumoural Immune Response in a Syngeneic Melanoma Model

As both EV groups increased OVA‐specific CD8^+^ T cells and their capacity to secrete IFNγ following EV treatment, we tested their effect on antigen‐expressing cancer cells in vivo. In both therapeutic and prophylactic models, subcutaneously (s.c.) injected OVA‐secreting B16 melanoma was used. These cells do not present OVA on their surface but instead display MHC:OVA peptide complexes, thereby directing the immune response toward adaptive T cell–mediated anti‐cancer activity while excluding antibody‐driven mechanisms. In all tumour models, mice with ulcerating tumours were excluded from flow cytometry, ELISA, and ELISpot analyses.

**FIGURE 4:**
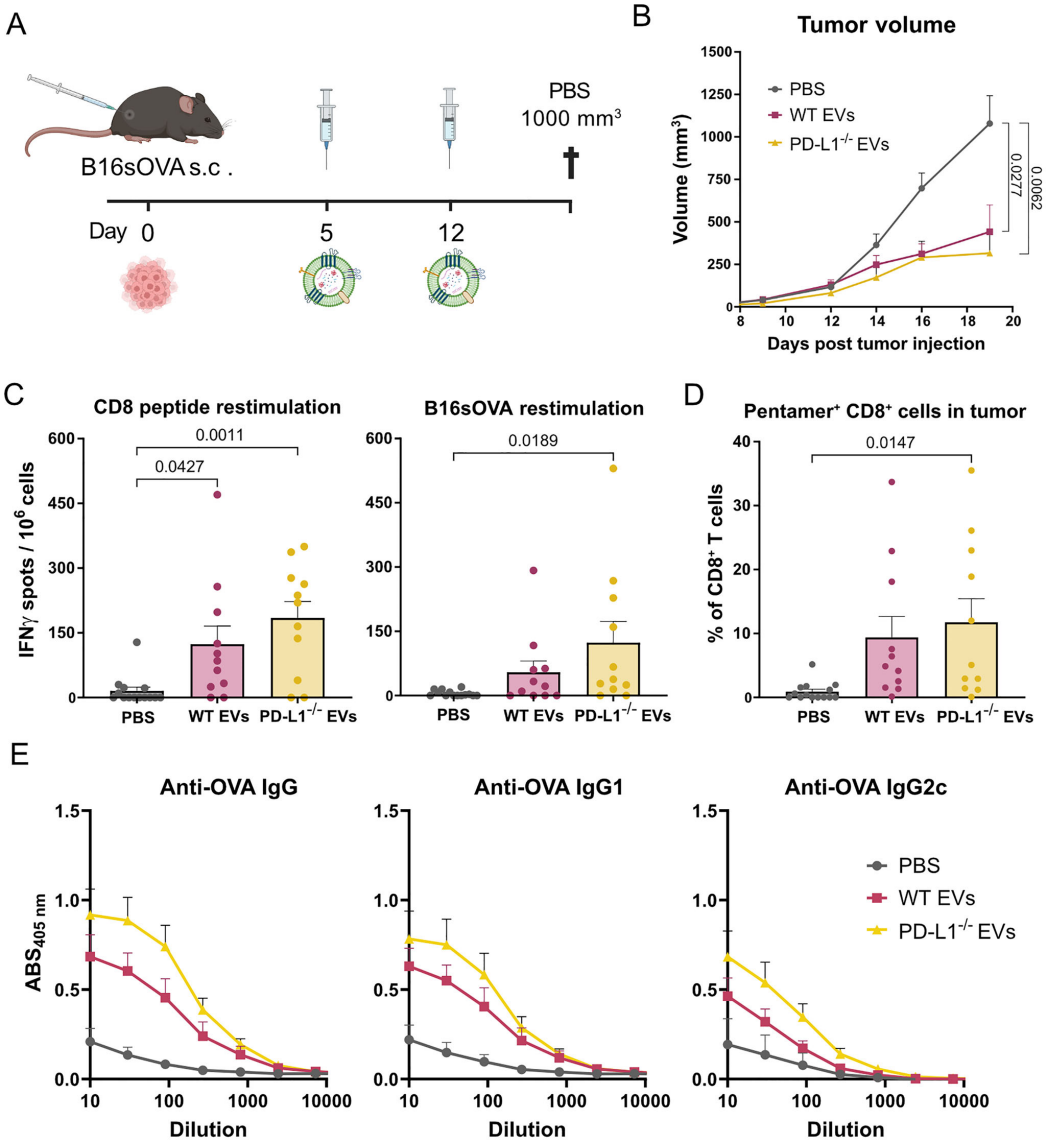
Therapeutic PD‐L1^−/−^ EVs induce a strong anti‐tumoural immune response in a syngeneic melanoma model. (A) Schematic representation of the tumour model with the same endpoint day: all mice were euthanized when the tumours in the PBS‐treated group reached 1000 mm^3^. (B) Tumour growth curves in mm^3^. (C) IFNγ ELISpot signal in splenocytes after restimulation with the OVA‐derived CD8 T cell peptide SIINFEKL or OVA‐secreting B16 tumour cells. (D) Frequency of OVA‐specific (Pentamer^+^) CD8^+^ T cells out of all CD8^+^ T cells (B220^−^CD3^+^CD8^+^) in the tumour, as assessed by flow cytometry. (E) ELISA for detection of OVA‐specific antibodies in serum. Data represents pooled data from two independent experiments (*n* = 12 biological replicates). Data are shown as mean ± SEM. Data were analysed using one‐way ANOVA with Tukey's test for multiple comparisons (B and C). Only statistically significant differences (*p* < 0.05) are indicated.

In the first tumour model, WT mice were s.c. injected with B16sOVA cells on day 0. On days 5 and 12, they received 40 µg of WT or PD‐L1^−/−^ EVs. All mice were sacrificed when >60% of PBS‐treated controls reached a tumour volume of 1000 mm^3^ (Figure 4A). This allows for a snapshot of the tumour and immune environment at the same time after tumour inoculation for each mouse.

By day 19, tumours in PBS‐treated mice had reached an average size of 1078.5 mm^3^, while those in EV‐treated mice remained significantly smaller, averaging 441.6 mm^3^ for WT EVs and 315.6 mm^3^ for PD‐L1^−/−^ EVs (Figure 4B). While both groups inhibited tumour growth, PD‐L1^−/−^ EVs showed a slight trend towards more pronounced inhibition, but with no statistical difference in relation to WT EVs. Splenocytes from both WT and PD‐L1^−/−^ EV treated mice demonstrated a high frequency of IFNγ secreting cells upon restimulation with the OVA‐peptide (*p* = 0.0427 and *p* = 0.0011, respectively; Figure 4C). No significant difference in IFNγ secretion was found between B16sOVA restimulated splenocytes of the WT EV and PBS control group (*p* = 0.4653). Interestingly, only PD‐L1^−/−^ EV treated mice responded significantly better to B16sOVA restimulation compared to PBS (*p* = 0.0189) as shown by ELISpot. Similarly, the frequency of OVA‐specific CD8^+^ T cells within tumours was significantly higher in PD‐L1^−/−^ EV‐treated mice compared to PBS controls (*p* = 0.0147), but not in WT EV‐treated mice (*p* = 0.0656, Figure 4D). Lastly, there were no significant differences in OVA‐specific IgG antibodies produced in the treatment groups.

PBS injection served as a non‐EV control. Notably, despite OVA being secreted by the tumour cells, the PBS group exhibited minimal immune activation compared to the EV‐treated groups (Figure 4C–E).

In conclusion, both EV groups were able to induce anti‐tumoural responses against the model antigen OVA. However, in comparison to PBS control, PD‐L1^−/−^ EVs induced a more robust anti‐tumoural response than WT EVs.

### 3.6 WT and PD‐L1^−/−^ Therapeutic EVs Increase Survival in a Syngeneic Melanoma Model, but Only PD‐L1^−/−^ EVs Increase Immune Cell Infiltration in the Tumour

**FIGURE 5:**
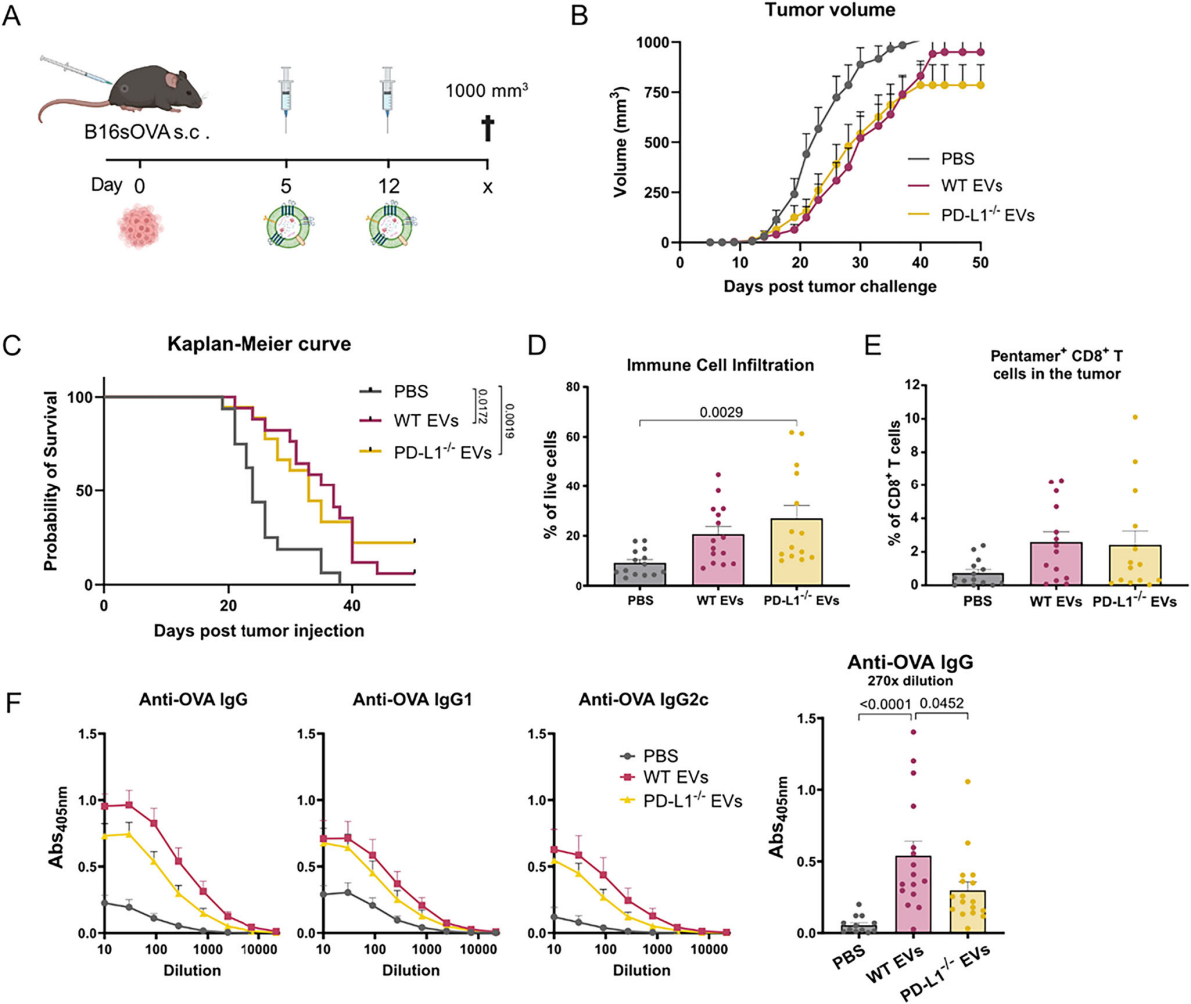
WT and PD‐L1^−/−^ therapeutic EVs increase survival in a syngeneic melanoma model, but only PD‐L1^−/−^ EVs increase immune cell infiltration in the tumour. (A) Schematic representation of the survival tumour model. Tumour growth was monitored, and mice were euthanized when their tumour reached 1000 mm^3^. (B) Tumour growth curves in mm^3^. (C) Kaplan‐Meier survival curves. (D) Frequency of CD45^+^ cells within tumours at analysis, as assessed by flow cytometry. (E) Frequency of OVA‐specific (Pentamer^+^) CD8^+^ T‐cells within CD8^+^ T cells (CD45^+^B220^−^CD3^+^CD8^+^) in the tumour at analysis, as assessed by flow cytometry. Data represents pooled data from 3 independent experiments, *n* = 16 biological replicates for PBS and PD‐L1^−/−^ EVs, *n* = 17 for WT EVs (B, C and F). *n* = 14 biological replicates for PBS, WT and PD‐L1^−/−^ EVs (D and E). Data are shown as mean ± SEM. Data were analysed by using log‐rank (Mantel‐Cox) test (C) and using one‐way ANOVA with Tukey's test for multiple comparisons (D, E and F). Only statistically significant differences (*p* < 0.05) are indicated.

In the second tumour model, mice received therapeutic injections as described above but were sacrificed individually on different days when their tumour reached 1000 mm^3^, to approximate a survival model (Figure 5A).

The tumour growth curve suggested that PD‐L1^−/−^ EVs exert a similar inhibitory effect on tumour progression as WT EVs (Figure 5B). Both EV treatments significantly improved survival compared to PBS control treatment (WT EVs *p* = 0.0019, and PD‐L1^−/−^ EVs *p* = 0.00172; Figure 5C). Interestingly, most mice that did not develop detectable tumours by the end of the experiment were part of the PD‐L1^−/−^ EV treatment group. The proportion of tumour‐free mice was 0% for PBS, 5.9% for WT EVs, and 22.2% for PD‐L1^−/−^ EVs. Furthermore, in comparison to PBS‐treated controls, a significant increase in intratumoural immune cell infiltration was only observed in PD‐L1^−/−^ EV‐treated mice (*p* = 0.0029; Figure 5D). Both EV groups showed a similar frequency of OVA‐specific CD8^+^ T cells in the tumour (Figure 5E). Notably, WT EV‐treated mice exhibited significantly higher levels of OVA‐specific IgG compared to the PD‐L1^−/−^ group (*p* = 0.0452; Figure 5F), a finding not observed in the immunization or initial cancer models. Lastly, to test if the anti‐tumourigenic effects of antigen‐loaded EVs were due to innate activation by the EVs, we repeated the experiment using EVs not loaded with antigen. No significant differences in tumour volume or survival were observed between the unloaded EV groups and PBS controls (Figure S4), supporting the antigen‐dependence of the therapeutic effect.

In conclusion, antigen‐loaded WT and PD‐L1^−/−^ EVs significantly enhanced survival in the therapeutic cancer model. PD‐L1^−/−^ EV‐treated mice exhibited increased immune cell infiltration within the tumour, while WT EVs induced significantly higher levels of OVA‐specific IgG in the serum.

### 3.7 In a Tumour Model With Prophylactic EV Administration, PD‐L1^−/−^ EVs Induce a Stronger Anti‐Tumoural Immune Response Than WT EVs

The above in vivo models showed limited differences between EV treatment groups, likely due to their short duration. The aggressive B16 model often reaches endpoints before a robust adaptive immune response develops. To address this, we employed a prolonged model to allow full immune maturation.

**FIGURE 6:**
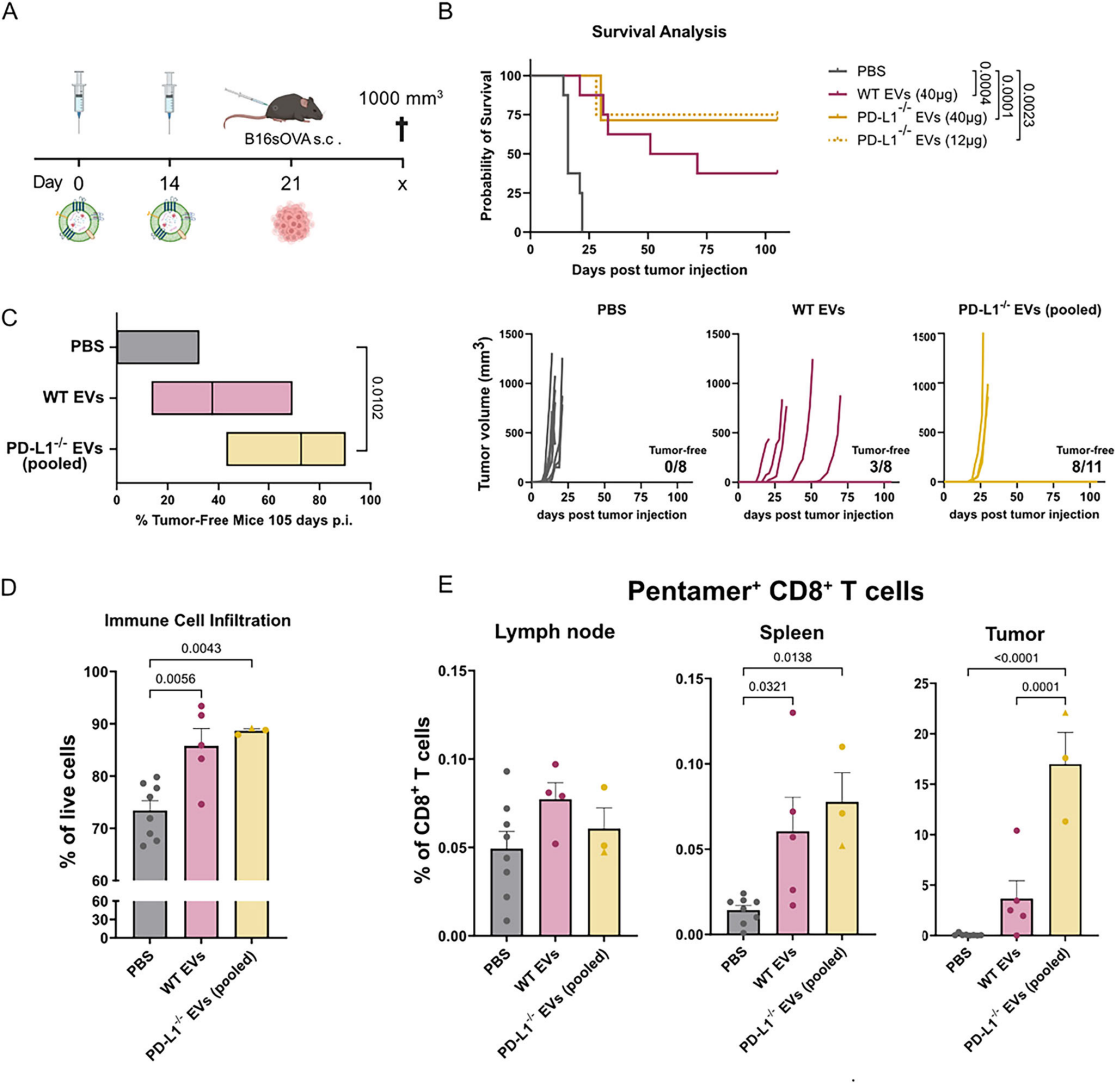
In a tumour model with prophylactic EV administration, PD‐L1^−/−^ EVs induce a stronger anti‐tumoural immune response than WT EVs. (A) Schematic representation of the tumour model with prophylactic EV administration. Tumour growth was monitored, and mice were euthanized when their tumour reached 1000 mm^3^. (B) Kaplan‐Meier survival curves (*n* = 8 biological replicates for PBS and WT, *n* = 7 for PD‐L1^−/−^ 40 µg, *n* = 4 for PD‐L1^−/−^ 12 µg, triangle symbol (D–E)) and individual tumour growth curves with number of tumour‐free mice out of all mice at 105 days post tumour injection. (C) Proportion and 95% confidence interval of tumour‐free mice at 105 days post tumour injection. (D) Frequency of CD45^+^ cells within tumours at analysis, as assessed by flow cytometry. (E) Frequency of OVA‐specific (Pentamer^+^) CD8^+^ T‐cells within CD8^+^ T cells (B220^−^CD3^+^CD8^+^) in lymph node, spleen and (CD45^+^B220^−^CD3^+^CD8^+^) tumour at analysis, as assessed by flow cytometry. Data are shown as mean ± SEM (A, B, D and E) and proportion with 95% confidence interval (C). Data were analysed using the log‐rank (Mantel‐Cox) test (B), Wilson/Brown method for 95% confidence interval and pairwise comparison using the Fisher's exact test with Benjamini‐Hochberg correction (C), and one‐way ANOVA with Tukey's test for multiple comparisons (D and E). Only statistically significant differences (*p* < 0.05) are indicated.

In the prophylactic tumour model, WT mice received 40 µg of WT or PD‐L1^−/−^ EVs on days 0 and 14, followed by s.c. injection of B16sOVA cells on day 21. Tumour growth was monitored, and mice were sacrificed individually once their tumours reached a volume of 1000 mm^3^ (Figure 6A). In the previous in vivo experiments, EV batches with comparable OVA content were used. However, in this model, such matched batches were unavailable. To account for differences in OVA levels, two doses (12 and 40 µg) of the same batch of PD‐L1^−/−^ EVs were administered. The 12‐µg dose was selected to match the OVA content of the 40 µg WT EV group. Interestingly, both PD‐L1^−/−^ EV doses resulted in similar survival outcomes (Figure 6B). Consequently, these groups were pooled in further analyses. At 105 days post‐tumour injection, a significantly higher proportion of mice in the PD‐L1^−/−^ EV group (8 out of 11 mice (72.7%), *p* = 0.0102) remained tumour‐free, compared to PBS controls which had all developed tumours (Figure 6C). This difference was not observed in the WT EV group (3 out of 8 mice (37.5%), *p* = 0.2000).

In the previous therapeutic experiment, a significant increase of immune cells in the tumour compared to PBS was observed in the PD‐L1^−/−^ EV group (Figure 5D). However, in this prophylactic tumour model, both EV groups showed similar immune infiltration in the tumour (Figure 6D). No overt differences in the composition of intratumoural immune cell populations were detected between the EV‐treated groups (Figure S5A). Flow cytometric analysis of OVA‐specific CD8^+^ T cells revealed no significant differences in their frequency within the tumour‐draining lymph nodes across groups (Figure 6E). In contrast, both EV‐treated groups showed elevated frequencies of OVA‐specific CD8^+^ T cells in the spleen compared to PBS controls. Most notably, within the tumour itself, PD‐L1^−/−^ EVs induced a significantly higher frequency of OVA‐specific CD8^+^ T cells out of all CD8^+^ T cells compared to both PBS and WT EV‐treated mice (*p* < 0.0001 and *p* = 0.0001). A similar pattern was observed when analysing the frequency of OVA‐specific CD8^+^ T cells among all live cells in the lymph node and spleen, and among live CD45^+^ cells in the tumour (*p* = 0.0005 and *p* = 0.0090; Figure S5B).

To assess whether EV treatment influenced tumour cell surface marker expression, we examined MHC‐I and PD‐L1 levels on tumour cells. WT EV treatment significantly upregulated both MHC‐I and PD‐L1 expression (*p* = 0.0106 and *p* = 0.0032); Figure S5C). In contrast, PD‐L1^−/−^ EV treatment led to a significant increase in PD‐L1 expression only (*p* = 0.0032), while the increase in MHC‐I expression did not reach statistical significance.

To evaluate whether tumour‐free mice had developed a robust OVA‐specific immune response, we analysed the frequency of OVA‐specific CD8^+^ T cells in the spleen and in the lymph, node draining the tumour injection site (Figure S5D). Although PD‐L1^−/−^ EV‐treated tumour‐free mice appeared to have higher frequencies of OVA‐specific CD8^+^ T cells in the draining lymph node compared to WT EV‐treated mice, this difference was not statistically significant. Notably, the frequency of OVA‐specific CD8^+^ T cells in the draining lymph node of PD‐L1^−/−^ EV treated tumour‐free mice reached 0.18%, compared to only 0.018% in tumour‐bearing mice (Figure S5B,D). Both groups exhibited similar frequencies of OVA‐specific CD8^+^ T cells in the spleen.

In conclusion, in a prophylactic tumour model, PD‐L1^−/−^ EVs elicited a more potent anti‐tumoural immune response than WT EVs, as evidenced by a significantly higher frequency of OVA‐specific CD8^+^ T cells within the tumour and a higher proportion of tumour‐free mice.

## 4 Discussion

Tumour‐derived EVs expressing the immune checkpoint molecule PD‐L1 have been shown to promote tumour progression by fostering an immunosuppressive microenvironment (Liu et al. 2022). In contrast, DC‐derived EVs loaded with tumour antigens are being investigated as therapeutic cancer vaccines (Besse et al. 2016; Escudier et al. 2005; Larssen et al. 2019; Markov et al. 2019; Morse et al. 2005; Veerman et al. 2023). However, EVs from activated DCs also express surface PD‐L1, potentially limiting their immunostimulatory capacity. In this study, we successfully generated PD‐L1 knockout EVs from BMDCs. These EVs retained key characteristics of WT BMDC EVs, including surface marker expression, size, morphology, in vitro cell‐binding and activation capacity. Moreover, in immune‐related proteome analysis, the lack of PD‐L1 was the strongest discerning factor between WT and PD‐L1^−/−^ EVs. Both WT and PD‐L1^−/−^ EVs induced OVA‐specific CD8^+^ T cells, enhanced immune infiltration, and delayed tumour growth in vivo. Notably, in relation to PBS, PD‐L1^−/−^ EVs elicited stronger immune responses across multiple assays, including higher frequencies of antigen‐specific CD8^+^ T cells, increased immune cell infiltration, and improved survival compared to WT EVs. The most pronounced effect was observed in a prophylactic tumour model, where PD‐L1^−/−^ EVs led to a greater proportion of tumour‐free mice and significantly elevated intratumoural OVA‐specific CD8^+^ T cell frequencies compared to WT EVs.

Given that MHC class I molecules on EVs play a relatively minor role in mediating in vivo effects (Hiltbrunner et al. 2016), which downplays the importance of direct antigen presentation, it was somewhat surprising that the absence of a co‐inhibitory molecule like PD‐L1 had such a strong impact on T cell activation and tumour control in vivo. Indeed, our in vitro data show that the EVs can have a direct effect on innate immune cells, with a stronger activation by PD‐L1 KO EVs. In addition, it is also possible that PD‐L1 on EVs directly engage with PD‐1 on CD8^+^ T cells to suppress their activation. PD‐1 expression has been described on DCs (Park et al. 2014), and in accordance with this we found PD‐1 expression at low levels on BMDCs (Figure 1A). PD‐1 on DCs has been shown to be associated with inhibitory signalling: blocking PD‐1/PD‐L1 interaction during DC maturation enhanced their survival (Park et al. 2014), and PD‐1‐deficient DCs were more potent at activating CD8^+^ T cells (Lim et al. 2016). Our findings underscore the importance of minimizing immune‐inhibitory molecules on therapeutic EVs, either through careful selection of the EV source or via engineering strategies.

A limitation of the tumour model used in this study is its rapid progression, which restricts the development of a robust adaptive immune response that typically requires ∼14 days (Pennock et al. 2013). In the initial model, mice were sacrificed at days 11 and 14 post‐EV injection, potentially limiting the detection of strong immune activation. Despite OVA secretion by the tumour, PBS‐treated controls showed minimal immune responses, unlike EV‐treated groups. This model offers a synchronized immune snapshot across groups. The survival model allowed more time for immune priming. Notably, WT EV‐treated mice exhibited elevated levels of OVA‐specific IgG antibodies, suggesting a stronger B cell response. In contrast, while all but one WT EV‐treated mice eventually developed tumours, multiple mice in the PD‐L1^−/−^ EV group remained tumour‐free. These findings support that the observed anti‐tumour immunity in this model is likely not mediated by antibodies, but rather by other immune mechanisms. While the survival model allowed more time for adaptive immune responses to develop, the aggressive tumour growth may have outpaced full effective immune activation. To address this, we employed a prophylactic model in which EVs were administered prior to tumour challenge. Given the minimal antigen‐specific activation in PBS controls, we consider this approach justified. Indeed, the difference in immune stimulation between WT and PD‐L1^−/−^ EVs was most striking in the prophylactic model. In this setting, the immune system has time to develop a strong, antigen‐specific T cell memory before the tumour challenge, leaving room for immunological benefits elicited by removal of PD‐L1 to manifest. Importantly, similar T cell responses observed in therapeutic models suggest that the tumour microenvironment does not fully suppress the EV‐induced immunity. However, we cannot exclude that differences observed between the survival and prophylactic models may be influenced by tumour‐derived PD‐L1^+^ EVs accumulating in secondary lymphoid organs in the therapeutic setting, where such tumour‐derived immunosuppressive signals are present already at the time of therapeutic EV administration. The absence of such signals at the time of vaccination is likely a contributor to the more effective EV‐mediated immune stimulation observed in the prophylactic model.

In the prophylactic tumour model, two PD‐L1^−/−^ EV doses were tested due to challenges in matching OVA content with WT EVs. One group received the same number of OVA but less total EV protein (12 µg), while the other received the same amount of total EV protein and thus more OVA (40 µg). Both groups showed similar immune responses and were merged to increase statistical power. This suggests that the lower dose may already reach the threshold for maximal response. Although prior titration data supported the use of 40 µg over 10 µg (data not shown), the EVs in the current study were further purified via TFF, potentially enhancing potency (Qazi et al. 2009). These findings highlight the need for dose optimization in EV therapies to balance efficacy with production efficiency.

Both EV‐treated groups enhanced immune cell infiltration, converting the tumours from immunologically ‘cold’ to ‘hot’. Despite analysing multiple immune populations, no significant differences in frequency or activation markers were found between EV groups. EV treatment also increased MHC‐I and PD‐L1 expression on tumour cells, likely driven by IFNγ from OVA‐specific CD8^+^ T cells (Blank et al. 2004). While MHC‐I upregulation improves tumour visibility to cytotoxic T cells, elevated PD‐L1 may suppress their function. However, it could also enhance responsiveness to checkpoint blockade. This aligns with our previous findings by Veerman et al. 2023, where antigen‐loaded EVs sensitized tumours to anti–PD‐1/PD‐L1 therapy, suggesting a potential strategy to overcome resistance in checkpoint‐refractory tumours.

Our findings demonstrate that the removal of PD‐L1 from antigen‐loaded DC‐derived EVs increases their capacity to induce immune activation. For clinical translation, autologous DCs with PD‐L1 knockout and immune adjuvants like αGC could be considered (Gehrmann et al. 2013). However, due to impaired immune function in many cancer patients, EVs from donor‐derived or off‐the‐shelf cell lines may be more feasible than autologous EVs. Prior work supports the immunogenicity of allogeneic EVs (Larssen et al. 2019). Thus, therapeutic EVs could be derived from a cell line that naturally or genetically engineered lacks PD‐L1 expression, and exogenously loaded with tumour antigens. Importantly, our proteomic analysis also indicates the presence of other EV‐associated immunomodulatory molecules, which may contribute to their function and present targets for future investigations. These findings highlight the need for continued optimization of EV engineering and delivery strategies, including cell line–derived EVs, hybridosomes, and EV‐mimetics (Zhou et al. 2025).

In conclusion, antigen‐loaded BMDC‐derived EVs are potent immune stimulators, and removal of immune checkpoint molecules such as PD‐L1 further enhances their immunogenicity. This removal could increase their potential in tumour immunotherapy. These findings also underscore the need for continued research into the functional role of EV cargo to fully unlock the therapeutic potential of EV‐based strategies.

## Author Contributions

**Loes Teeuwen**: methodology, investigation, visualization, formal analysis, writing – original draft, writing – review and editing. **Loïc Steiner**: methodology, investigation, visualization, formal analysis, writing – original draft, writing – review and editing. **Chantal Reinhardt**: methodology, investigation, visualization, formal analysis, writing – original draft, writing – review and editing. **Annemarijn Offens**: investigation, writing – review and editing. **Jesse E. Kuipers**: investigation, writing – review and editing. **Daniel Martínez‐Martínez**: investigation, writing – review and editing. **Jules Mazouin**: investigation, writing – review and editing. **Benedict J. Chambers**: conceptualization, resources, writing – review and editing. **Gözde Güçlüler Akpinar**: conceptualization, methodology, investigation, writing – review and editing. **Susanne Gabrielsson**: conceptualization, methodology, funding acquisition, resources, project administration, supervision, writing – review and editing

## Conflicts of Interest

Susanne Gabrielsson has a patent on B cell derived exosomes in immune therapy. The other authors declare no conflicts of interest.

## Supporting information




**Supporting Information**: jev270322‐sup‐0001‐SuppMat.docx

## Data Availability

The data that support the findings of this study are available from the corresponding author upon reasonable request.
