# Supplementary material for: Eliminating PD‐L1 on Dendritic Cell Extracellular Vesicles for Immunotherapy Potentiates Immune‐Mediated Tumour Rejection in Mice
Source: J Extracell Vesicles. 2026 Jun 30;15(7):e70322. doi: 10.1002/jev2.70322 (PMC13317667; doi:10.1002/jev2.70322)
Supplement: Supplementary file 1 — Supporting Information: jev270322‐sup‐0001‐SuppMat.docx [file JEV2-15-e70322-s001.docx]

**
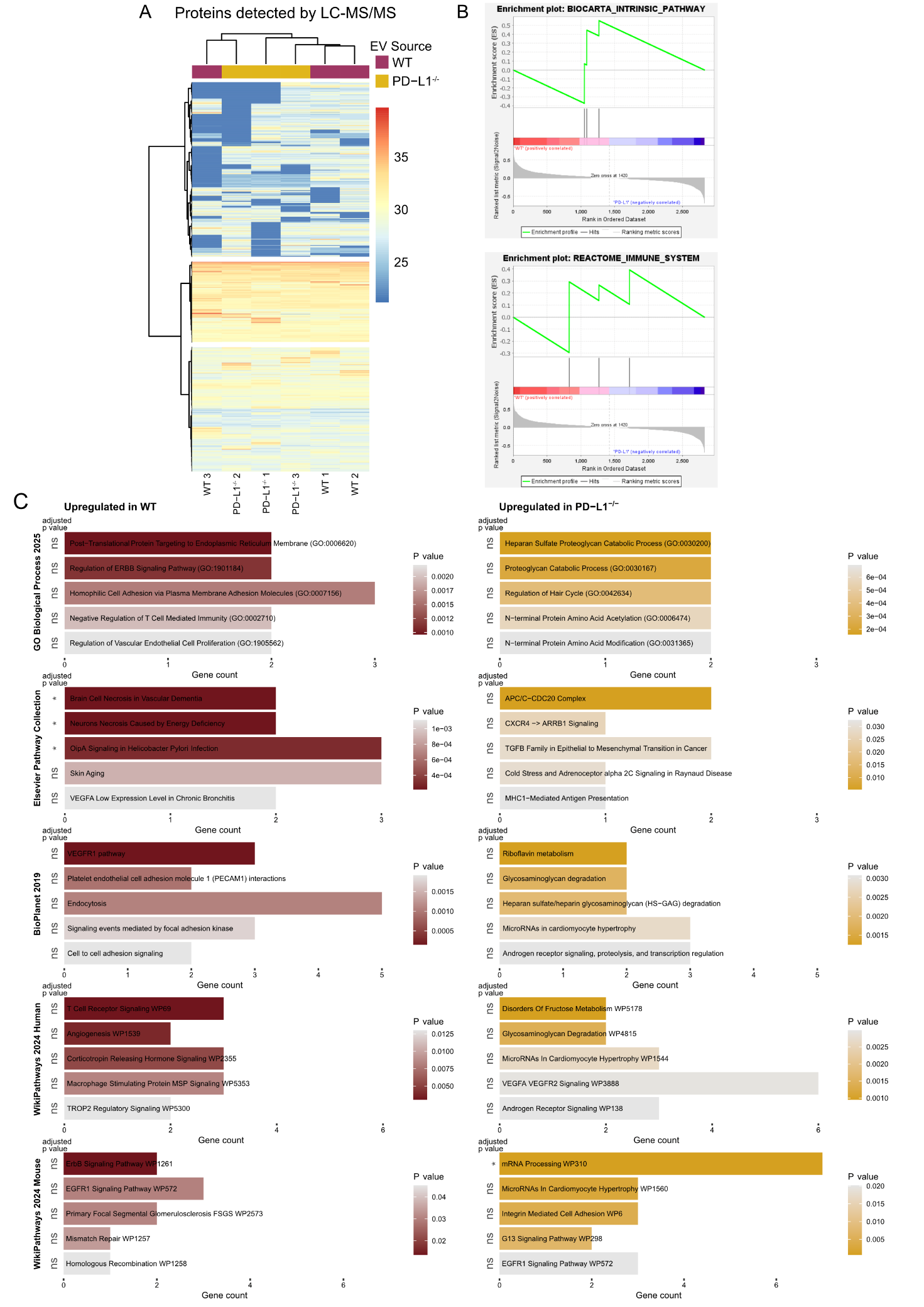
**

**Supplementary Figure 1. Proteomic Analysis of WT and PD-L1^-/-^ EVs show similar proteomic composition.** A) Heatmap with hierarchical clustering of all detected proteins. B) Enrichment plots for two exemplary pathways (Intrinsic pathway from Biocarta and Immune System from Reactome) showing no significant enrichment. C) EnrichR-based analysis of significantly differentially abundant proteins show no significant enrichment in immune-related terms in GO-Terms of biological processes, the Elsevier pathway collection, BioPlanet and human and mouse WikiPathways.


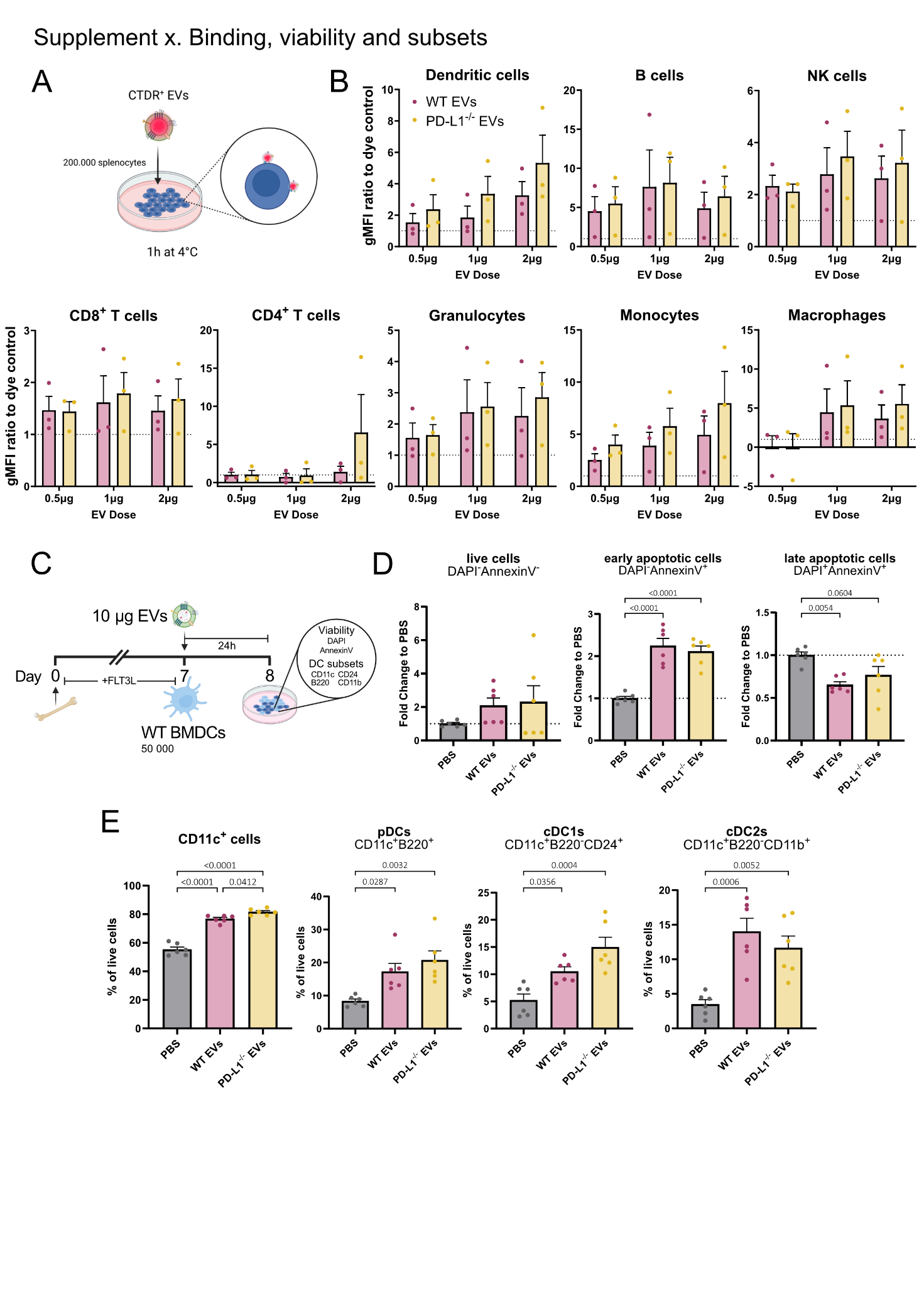


**Supplementary Figure 2. WT and PD-L1^-/-^ EVs show comparable splenocyte binding and effects on BMDC viability and subset distribution**. A) Schematic representation of binding experiment. B) Geometric mean of fluorescent intensity (gMFI) ratio of different doses of CellTracker^TM^ Deep Red-labelled (CTDR^+^) EVs on various splenocytes divided by dye only control (n = 3 independent biological replicates of EVs), as assessed by flow cytometry. Cells were gated as follows: Dendritic cells (B220^-^CD4^-^CD8^-^F4/80^-^NK1.1^-^CD11c^+^MHCii^hi^), B cells (B220^+^), NK cells (B220^-^CD3^-^NK1.1^+^), CD8^+^ T cells (B220^-^CD3^+^CD4^-^CD8a^+^), CD4^+^ T cells (B220^-^CD3^+^CD8a^-+^CD4^+^), Granulocytes (B220^-^TCRβ^-^CD11b^+^Ly6C^+^Ly6G^+^), Monocytes (B220^-^TCRβ^-^CD11b^+^Ly6C^+^Ly6G^-^) and Macrophages (B220^-^TCRβ^-^CD11b^+^Ly6C^-^F4/80^+^). C) Schematic representation of Flt3L-differentiated BMDC activation. D) Frequencies of viable, early and late apoptotic BMDCs after 24h incubation with PBS or 10 µg WT or PD-L1^-/-^ EVs, as assessed by flow cytometric analysis of DAPI and AnnexinV staining. E) Frequencies of CD11c^+^ cells and of phenotypically defined dendritic cell subsets (plasmacytoid [pDC], conventional type 1 [cDC1] and conventional type 2 [cDC2]), as assessed by flow cytometry. Data are pooled from two independent experiments (D and E). Data are shown as mean +/- SEM. Data were analyzed by using two-way ANOVA with Šídák's (B) or Tukey’s (D and E) test for multiple comparisons. Only statistically significant differences (p < 0.05) are indicated.


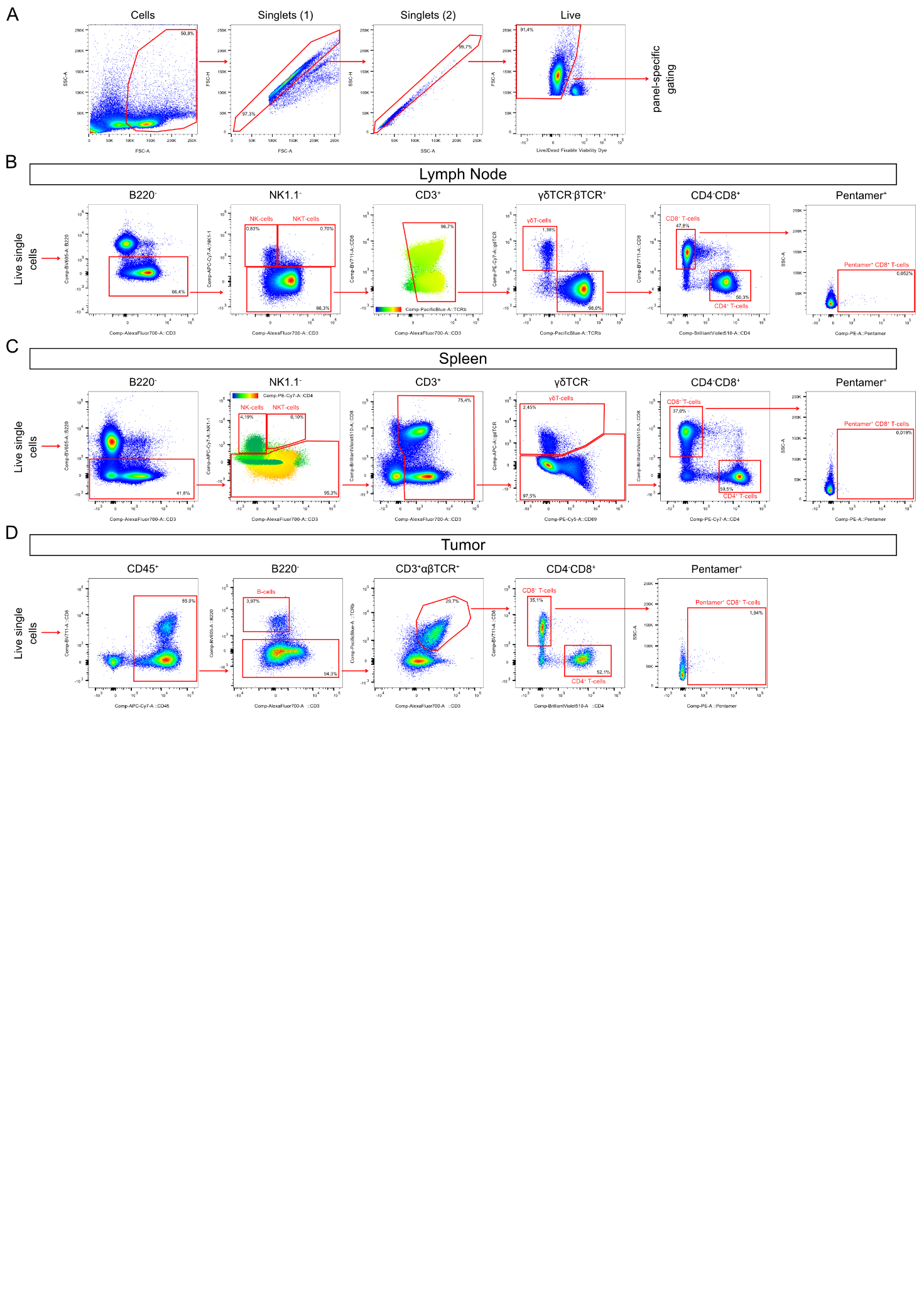


**Supplementary Figure 3. Gating strategy for live Pentamer^+^ CD8^+^ T cells.** Gating strategy for A) single live cells and, subsequently, pentamer^+^ CD8^+^ T cells in B) Lymph node, C) Spleen and D) Tumor.


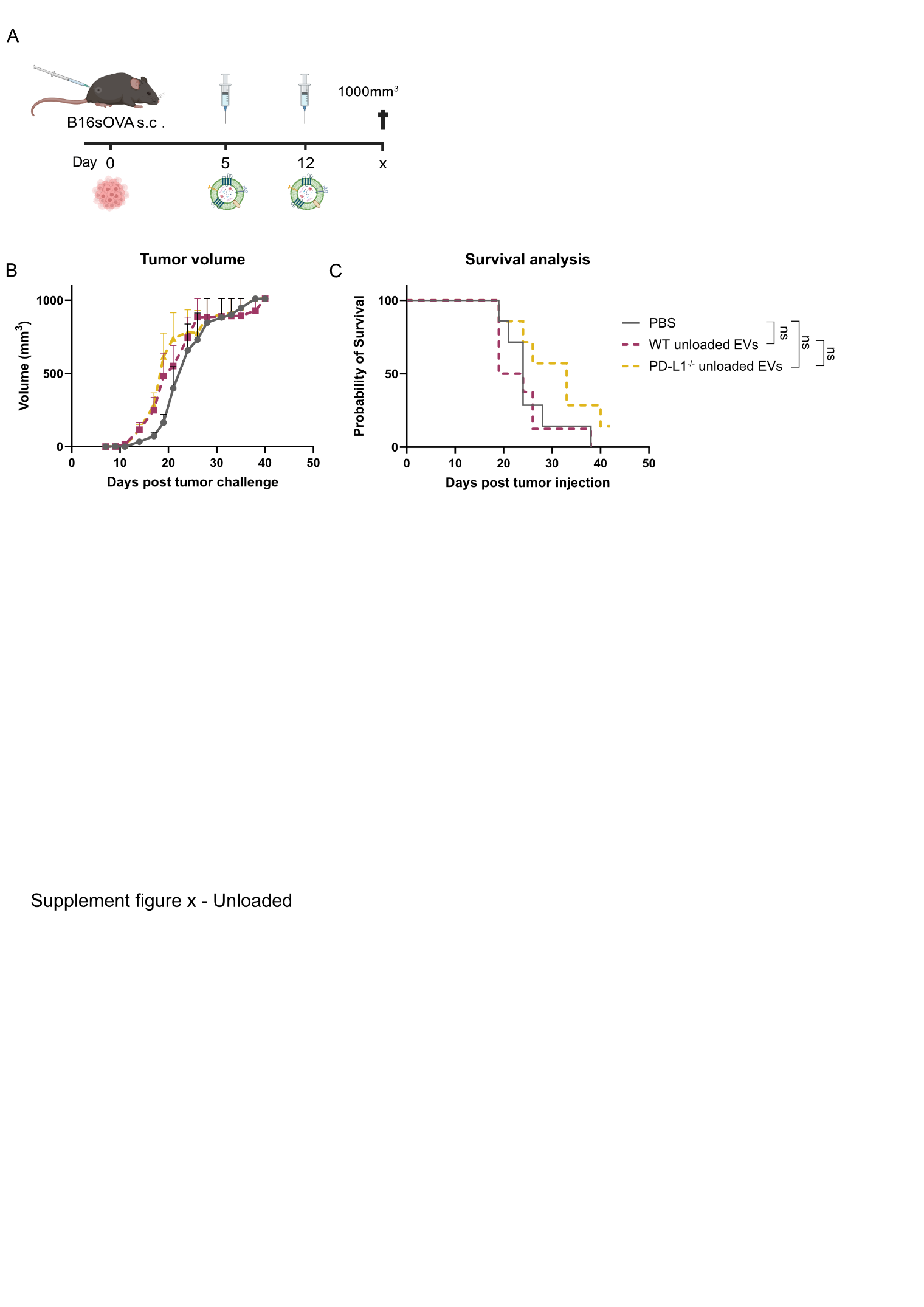


**Supplementary Figure 4. Unloaded therapeutic WT and PD-L1^-/-^ EVs do not increase survival in a syngeneic melanoma model.** A) Schematic representation of the survival tumor model. Tumor growth was monitored, and mice were euthanized when their tumor reached 1000mm^3^. B) Tumor growth curves in mm^3^. C) Kaplan-Meier survival curves. Data from 1 experiment. n = 6-8 biological replicates. Data are shown as mean +/- SEM. Data were analyzed by using log-rank (Mantel-Cox) test.


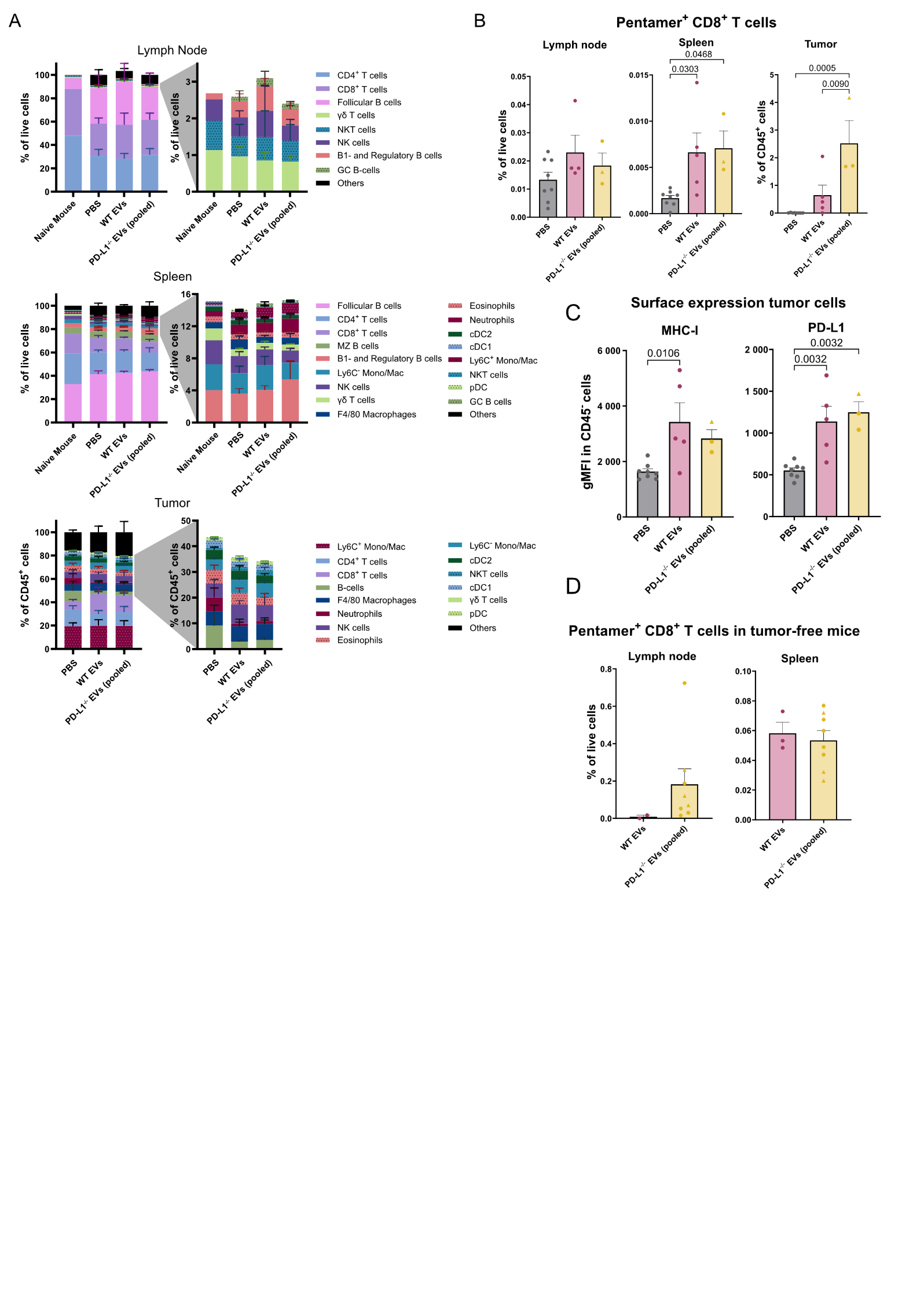


**Supplementary Figure 5. In a tumor model with prophylactic EV administration, PD-L1^-/-^ EVs induce a stronger anti-tumoral immune response than WT EVs.** A) Immune cell composition in lymph node, spleen and tumors at analysis, as assessed by flow cytometry. B) Frequency of OVA-specific (B220^-^CD3^+^CD8^+^Pentamer^+^) CD8^+^ T-cells within live cells in lymph node, spleen and (CD45^+^B220^-^CD3^+^CD8^+^ Pentamer^+^) in tumor at analysis, as assessed by flow cytometry. C) Geometric mean of fluorescent intensity (gMFI) of MHC-I and PD-L1 in CD45^-^ cells within tumors at analysis, as assessed by flow cytometry. D) Frequency of OVA-specific CD8^+^ T-cells (B220^-^CD3^+^CD8^+^Pentamer^+^) within live cells in lymph node and spleen in mice that were tumor-free at 105 days post tumor injection, as assessed by flow cytometry. Data are shown as mean +/- SD (A) and +/- SEM (B-D). Data were analyzed using one-way ANOVA with Tukey’s test for multiple comparisons (B-C) and unpaired T test (D). Only statistically significant differences (p < 0.05) are indicated.

**Supplementary Table 1**

|  | **Marker** | **Fluorophore** | **Clone** | **Catalog number** | **Company** |
| --- | --- | --- | --- | --- | --- |
| **BMDC & EVs** | CD11b | FITC | M1/70 | 101206 | Biolegend |
|  | CD11c | FITC | N418 | 117306 | Biolegend |
|  | CD14 | FITC | Sa14-2 | 123308 | Biolegend |
|  | Ham IgG | FITC | HTK888 | 400902 | Biolegend |
|  | PD-1 | FITC | 29F.1A12 | 135214 | Biolegend |
|  | Rat IgG1 | FITC | RTK2071 | 400405 | Biolegend |
|  | Rat IgG2a | FITC | RTK2758 | 400505 | Biolegend |
|  | Rat IgG2b | FITC | RTK4530 | 400605 | Biolegend |
|  | CD1d | PE | 1B1 | 123510 | Biolegend |
|  | CD40 | PE | 3/23 | 124610 | Biolegend |
|  | CD54 | PE | 3 e2 | 553253 | BD Biosciences |
|  | CD63 | PE | NVG-2 | 143904 | Biolegend |
|  | CD80 | PE | 16-10A1 | 104708 | Biolegend |
|  | CD81 | PE | Eat-2 | 104905 | Biolegend |
|  | CD83 | PE | Michel-19 | 558205 | BD Biosciences |
|  | CD86 | PE | GL-1 | 105008 | Biolegend |
|  | CD9 | PE | MZ3 | 124806 | Biolegend |
|  | CTLA4 | PE | UC10-4B9 | 106305 | Biolegend |
|  | Ham IgG | PE | HTK888 | 400904 | Biolegend |
|  | MHCI (Kb) | PE | AF6-88.5 | 116508 | Biolegend |
|  | MHC-II (I-A/I-E) | PE | M5/114.15.2 | 107608 | Biolegend |
|  | MHC-I-SIINFEKL | PE | 25-D1.16 | 141604 | Biolegend |
|  | Mouse IgG1 | PE | MOPC-21 | 400111 | Biolegend |
|  | Mouse IgG2a | PE | MOPC-173 | 400211 | Biolegend |
|  | PD-L1 | PE | 10F.9G2 | 124308 | Biolegend |
|  | PD-L2 | PE | TY25 | 107205 | Biolegend |
|  | Rat IgG2a | PE | RTK2758 | 400505 | Biolegend |
|  | Rat IgG2b | PE | RTK4530 | 400607 | Biolegend |
|  | PD-1 | PE | 29F.1A12 | 135206 | Biolegend |
|  |  |  |  |  |  |
| **Splenocyte** | MHC-II | BV510 | M5/114.15.2 | 107635 | Biolegend |
| **Binding** | CD45R/B220 | PB | RA3-6B2 | 103227 | Biolegend |
|  | CD8 | BV510 | 53-6.7 | 100752 | Biolegend |
|  | CD4 | APC-Cy7 | RM4-5 | 100526 | Biolegend |
|  | F4/80 | APC-Cy7 | BM8 | 123116 | Biolegend |
|  | NK1.1 | PerCPCy5.5 | PK136 | 108728 | Biolegend |
|  | CD11c | FITC | N418 | 117306 | Biolegend |
|  | CD3 | AF700 | 17A2 | 100216 | Biolegend |
|  | CD11b | BV786 | M1/70 | 101243 | Biolegend |
|  | Ly6G | PE-Cy7 | 18A | 127618 | Biolegend |
|  | Ly6C | PB | HK1.4 | 128014 | Biolegend |
|  |  |  |  |  |  |
| **BMDC Activation** | AnnexinV | APC-Fire 750 | - | 640953 | Biolegend |
|  | CD11c | FITC | N418 | 117306 | Biolegend |
|  | CD11b | PerCP-Cy5.5 | M1/70 | 101228 | Biolegend |
|  | CD24 | PE-Dazzle 594 | M1/69 | 101837 | Biolegend |
|  | B220 | PB | RA3-6B2 | 103227 | Biolegend |
|  | MHC-II (IA-IE) | BV785 | M5/114.15.2 | 107645 | Biolegend |
|  | MHC-I | BV510 | AF6-88.5 | 116523 | Biolegend |
|  | CD80 | BV650 | 16-10A1 | 104732 | Biolegend |
|  | CD86 | APC | GL-1 | 105012 | Biolegend |
|  | CD40 | PE-Cy7 | 3/23 | 124622 | Biolegend |
|  |  |  |  |  |  |
| **OT-I T Cell** | CD45.1 | BUV395 | A20 | 17816102 | Invitrogen |
| **Activation** | CD8a | APC-Cy7 | 53-6.7 | 100714 | Biolegend |
|  | CD25 | PerCP-Cy5.5 | PC61 | 102030 | Biolegend |
|  | CD44 | BV785 | IM7 | 103059 | Biolegend |
|  | CD69 | PE-Cy5 | H1.2F3 | 104510 | Biolegend |
|  |  |  |  |  |  |
| **Lymph Node** | CD45R/B220 | BV605 | RA3-6B2 | 103212 | Biolegend |
|  | CD3 | AF700 | 17A2 | 100216 | Biolegend |
|  | NK1.1 | APC-Cy7 | PK136 | 108724 | Biolegend |
|  | CD8a | BV711 | 53-6.7 | 100714 | Biolegend |
|  | CD4 | BV510 | RM4-5 | 100526 | Biolegend |
|  | TCRβ | PB | H57-597 | 109226 | Biolegend |
|  | γδTCR | PE-Cy7 | GL3 | 118116 | Biolegend |
|  | Pentamer | PE | — | F093-2B-E-93-H-2Kb | PROIMMUNE |
|  |  |  |  |  |  |
| **Spleen** | CD45R/B220 | BV605 | RA3-6B2 | 103212 | Biolegend |
|  | CD3 | AF700 | 17A2 | 100216 | Biolegend |
|  | NK1.1 | APC-Cy7 | PK136 | 108724 | Biolegend |
|  | CD8a | BV510 | 53-6.7 | 100714 | Biolegend |
|  | γδTCR (TCRGD) | APC | GL3 | 118116 | Biolegend |
|  | CD69 | BV510 | HI1.2F3 | 104531 | Biolegend |
|  | CD4 | PE-Cy7 | RM4-5 | 100526 | Biolegend |
|  | Pentamer | PE | — | F093-2B-E-93-H-2Kb | PROIMMUNE |
|  |  |  |  |  |  |
| **Tumor** | CD45 | APC-Cy7 | 30-F11 | 103116 | Biolegend |
|  | CD8a | BV711 | 53-6.7 | 100714 | Biolegend |
|  | CD45R/B220 | BV605 | RA3-6B2 | 103212 | Biolegend |
|  | CD3 | AF700 | 17A2 | 100216 | Biolegend |
|  | TCRβ | PB | H57-597 | 109226 | Biolegend |
|  | CD4 | BV510 | RM4-5 | 100526 | Biolegend |
|  | Pentamer | PE | — | F093-2B-E-93-H-2Kb | PROIMMUNE |
